# Copy number of 8q24.3 drives HSF1 expression and patient outcome in cancer: an individual patient data meta-analysis

**DOI:** 10.1186/s40246-019-0241-3

**Published:** 2019-11-07

**Authors:** Nele Brusselaers, Karl Ekwall, Mickael Durand-Dubief

**Affiliations:** 1 Centre for Translational Microbiome Research (CTMR), Department of Microbiology, Tumor and Cell biology, Karolinska Institutet, Karolinska Hospital, SE-17176 Stockholm, Sweden; 2 Science for Life Laboratory (SciLifeLab), SE-17165 Stockholm, Sweden; 3 Department of Biosciences and Nutrition, Karolinska Institutet, Neo building, Blickagången 16, S-141 52 Huddinge, Sweden

**Keywords:** Copy number alteration, 8q24.3, HSF1, Individual patient data meta-analysis, Patient outcome

## Abstract

**Background:**

The heat-shock transcription factor 1 (HSF1) has been linked to cell proliferation and survival in cancer and has been proposed as a biomarker for poor prognosis. Here, we assessed the role of HSF1 expression in relation to copy number alteration (CNA) and cancer prognosis.

**Methods:**

Using 10,287 cancer genomes from The Cancer Genome Atlas and Cbioportal databases, we assessed the association of HSF1 expression with CNA and cancer prognosis. CNA of 8q24.3 was categorized as diploid (reference), deletion (fewer copies), gain (+ 1 copy) and amplification (≥ + 2 copies). Multivariate logistic regression modeling was used to assess 5-year survival among those with a first cancer diagnosis and complete follow-up data (*N* = 9568), categorized per anatomical location and histology, assessing interaction with tumor stage, and expressed as odds ratios and 95% confidence intervals.

**Results:**

We found that only 54.1% of all tumors have a normal predicted 8q24.3 copy number and that 8q24.3 located genes including HSF1 are mainly overexpressed due to increased copies number of 8q24.3 in different cancers. The tumor of patients having respectively gain (+ 1 copy) and amplification (≥ + 2 copies) of 8q24.3 display a global increase of 5-year mortality (odds ratio = 1.98, 95% CI 1.22–3.21) and (OR = 2.19, 1.13–4.26) after full adjustment. For separate cancer types, tumor patients with 8q24.3 deletion showed a marked increase of 5-year mortality in uterine (OR = 4.84, [2.75–8.51]), colorectal (OR = 4.12, [1.15–14.82]), and ovarian (OR = 1.83, [1.39–2.41]) cancers; and decreased mortality in kidney cancer (OR = 0.41, [0.21–0.82]). Gain of 8q24.3 resulted in significant mortality changes in 5-year mortality for cancer of the uterus (OR = 3.67, [2.03–6.66]), lung (OR = 1.76, [1.24–2.51]), colorectal (OR = 1.75, [1.32–2.31]) cancers; and amplification for uterine (OR = 4.58, [1.43–14.65]), prostate (OR = 4.41 [3.41–5.71]), head and neck (OR = 2.68, [2.17–3.30]), and stomach (OR = 0.56, [0.36–0.87]) cancers.

**Conclusions:**

Here, we show that CNAs of 8q24.3 genes, including HSF1, are tightly linked to 8q24.3 copy number in tumor patients and can affect patient outcome. Our results indicate that the integration of 8q24.3 CNA detection may be a useful predictor for cancer prognosis.

## Background

Early diagnosis and accurate prognostic markers of cancer help practitioners in treatment decisions to ultimately optimize patient outcomes. Despite the advancements in diagnostic methods and the use of molecular diagnostics, for example, next-generation sequencing panels run in routine in an increasing number of laboratories for cancer patients [1, 2], clinical prognostics are often limited to histology, positivity of lymph nodes, and presence of metastases [3, 4]. In the light of personalized medicine, there is a need to explore feasible and reliable new biomarkers to improve prognostic information [5].

Recent studies have raised interest in the heat-shock transcription factor 1 (HSF1), master regulator of cell stress response for adaptation and survival [6]. When activated, HSF1 facilitates the transcription of genes, such as the heat shock proteins (HSPs) chaperones required to relieve the proteotoxic stress that can cause cell death [6]. Overexpression of HSF1 has been linked with cancer proliferation, and malignancy, suggesting that HSF1 could serve as a prognostic marker [7, 8]. Numerous clinical and basic research studies showed that high expression level of HSF1 is associated with poor outcomes in many cancer types [7–12], pointing out the potential of HSF1 as a prognostic biomarker [12, 13].

Nevertheless, the origin and the interpretation of HSF1 overexpression in cancer are poorly understood since HSF1 appears to drive a distinct regulation in cancer cells [8]. So far, consensus suggests that HSF1 overexpression helps to relieve the stress of protein unbalances [10, 14], likely caused by aneuploidy or an imbalanced karyotype [15–17]. Intriguingly, overexpression of HSF1 cancer signature gene clusters at the end of chromosome 8q [18]. However, mechanisms that drive HSF1 overexpression in different cancers remain largely unknown but may hold a key in understanding tumor development and the relationship to survival.

Clinical studies have now emerged with transcriptomic, genomic, and clinical patient data offering unprecedented opportunities to understand the molecular events associated with cancer, and its related outcome [19]. Gene expression seems to exhibit different expression profiles in various human cancer types [20]. In addition, acquired copy number alteration (CNA) in cancer cells is common [21] and can play a significant role in cancer development by altering gene dosage and affecting the expression of multiple genes, and regulatory regions [22–24].

The aim of this study using an individual patient data meta-analysis approach is to assess the overall role of HSF1 expression in relation to CNA in cancer prognosis.

## Methods

### Search strategy and selection criteria

This study used data from cBioportal portal (http://www.cbioportal.org) [18, 19], which includes peer-reviewed studies, METABRIC data (Molecular Taxonomy of Breast Cancer International Consortium), and unpublished data from The Cancer Genome Atlas (TCGA) [25, 26]. A descriptive summary of all data extracted from cBioportal on the acquired CNA and RNA expression per cancer type is presented in Additional file 1: Table S1.

For the survival analyses, only individuals without a prior history of cancer, and with identical CNA for the genes present in the 8q24.3 region (i.e., patient with heterogeneous CNA were excluded), as well the 5-year survival information, were included.

### Data extraction

Data extraction and genomic analyses were conducted by MDD and data management, and individual patient data meta-analyses by NB. Demographics, clinical information, and cancer genomics datasets were extracted for all individuals. Normalized mRNA expression data (*Z*-scores 2.0) were computed for the relative expression of an individual gene and tumor to the gene of the expression distribution compared to the reference population diploid for the corresponding gene (by default for mRNA), or normal samples (when specified)(http://www.cbioportal.org/faq.jsp). For CNA categories, data were obtained from Cbioportal [25, 26] and derived from Affymetrix SNP6 data (copy number ratio from tumor samples minus ratio from the matched normal tissues) computed with the GISTIC 2.0 algorithm [27].

The estimated copy number alteration of the 8q24.3 region was categorized according to the predicted copy number: deep deletion (− 2) (0.1%), shallow deletion (− 1) (4.7%), diploid or normal (0) (54%), gain (+ 1) (32%), and amplification (≥ + 2) (8.6%).

The main outcome in the individual patient data meta-analysis was the 5-year mortality (dead or alive) since the exact number of days of survival was only reported for 22% of the cohort and the secondary outcome was the risk of being alive, and healthy or not (to assess the combined effect of recurrence and mortality). The following data were collected and categorized: sex (categorized as male or female), age at time of diagnosis (categorized as < 40, 40–49, 50–59, 60–69, and ≥ 70 years), anatomical location and histological subtype, HSF1-expression (categorized in quartiles), tumor stage (categorized as stage 0–1 or in situ, stage II, stage III, stage IV), calendar period (categorized as 1978–2005, 2006–2008, 2009–2010, 2011–2013), study (42 different studies), history of any cancer (yes or no), and 5-year outcome (alive with or without recurrence, or dead). Missing values were crosschecked with other relevant variables. Length of follow-up and length of survival were missing for the majority of individuals, and therefore not used for survival modeling (only to complete missing data on the outcomes). Data on body mass index, smoking, alcohol-use, cancer-specific risk factors, and treatment were missing in the majority of the individuals or too heterogeneous among cancer types and were therefore not included.

### Data analysis

To avoid bias due to heterogeneous gene expression of HSF1 across various cancers (Additional file 1: Appendix 2), we analyzed co-expression using the Spearman correlation test generated from cBioportal. JMP® v13 (SAS Institute) and Tableau desktop® 10.5 (Tableau Software) were used for data processing and visualization. Gene ontology analysis was performed using Panther v12.0 [28].

Individual patient data meta-analyses were conducted in Stata/MP14.2 (StataCorp) using two methods to assess 5-year mortality and healthy survival overall and for each anatomical location and histological subtype [29]. Differences in descriptive statistics were compared by means of chi-square tests, with *p* values < 0.05 representing statistically significant differences. All results were expressed as odds ratios (OR) and 95% confidence intervals (CI) using diploidy as reference. If the odds ratio of 1 (indicating no difference) is included in the 95% confidence interval, the results do not indicate statistically significant differences between both groups. The first approach was based on random effect modeling using the ipdmetan package in Stata, which is a two-stage individual patient data meta-analysis pooling and visualizing the effect of binary outcomes by means of forest plots [30]. *I*^2^ statistics were used to quantify statistical heterogeneity, with values < 50%, 50–75%, and > 75% defined as low, moderate, and high heterogeneity, respectively [31]. Results were weighted by anatomical location, histological subtype, and study. Since this approach did not allow adjustment for confounding or interaction, multivariate logistic regression analyses were also conducted (one-step approach) [29]. For each anatomical location, three models were presented to compare four risk groups: diploid (reference), shallow/deep deletion (combined), gain, and amplification. Model 1 was unadjusted, model 2 was adjusted for sex, age, and calendar period and clustering by study, and model 3 was additionally adjusted for HSF1 expression and interaction with tumor stage. Interaction with tumor stage and HSF1 was assessed by likelihood-ratio testing. For histological subtypes, 8q24.3 CNA gain and amplification were combined into one category to increase power, and only models 1 and 2 were presented. Subgroup analyses distinguishing between gain and amplification were only conducted for the 15 histological subtypes with the highest number of individuals with gain or amplification. Analyses were only presented if at least 10 individuals were included in each risk group and are based on complete-case analyses.

## Results

### HSF1 expression profile across different cancers type

**Fig. 1:**
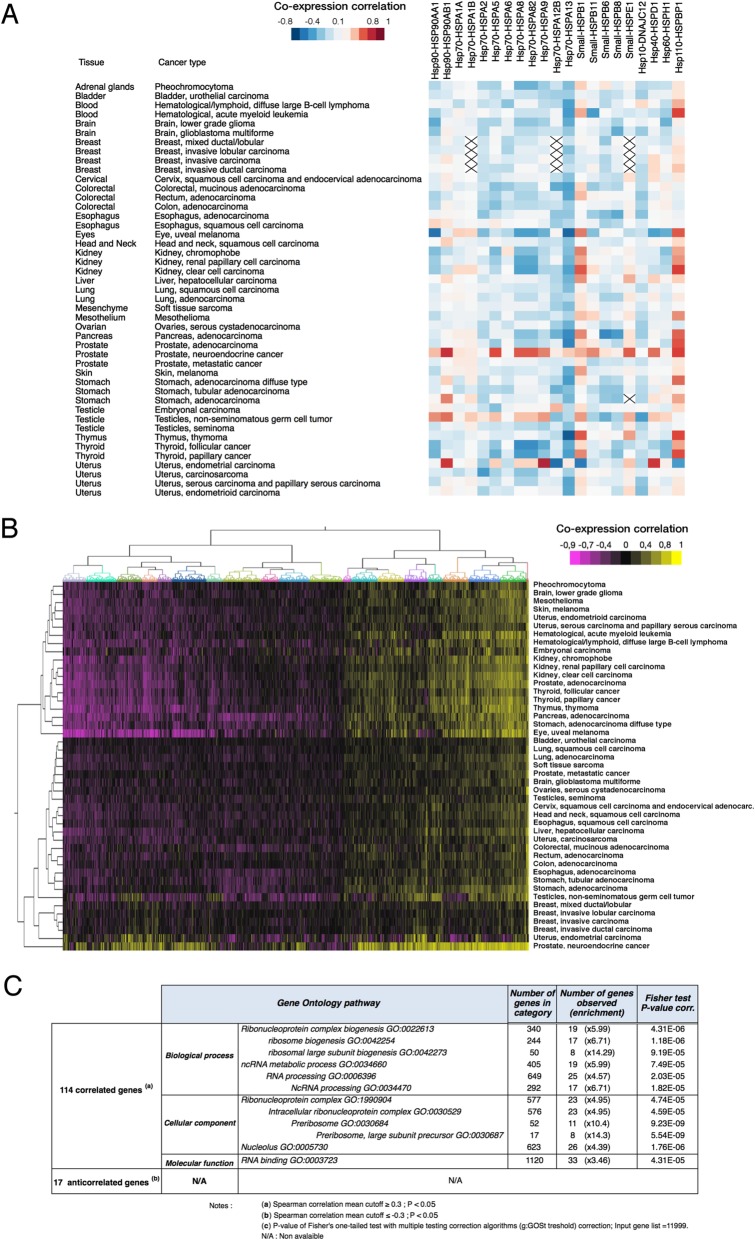
Expression of HSF1 in cancer is globally linked to the expression of genes involved in ribosomal biogenesis and not HSPs family genes. **a** Heatmap correlation of HSP gene family expression with HSF1 in different cancer types. *X* indicates no available data. **b** Hierarchical clustering for all gene expression with HSF1 in different cancer types. **c** Gene ontology analysis for the most positively and negatively expressed genes with HSF1 (Spearman’s correlation value cut-off ≥ 0.3 and cut-off ≤ − 0.3, *p* < 0.01). The number of genes in the category represents the genes identified in the Gene Ontology pathway. The number of genes observed shows the number of a gene correlated with HSF1 presents in the corresponding Gene Ontology category and its relative over-representation enrichment

In total, 11,069 patients were included with information on CNA and RNA expression. In none of the 45 histopathological cancer types, HSF1 expression displayed obvious linkage with HSPs genes (Fig. 1a). When looking at the whole expression level, hierarchical clustering presented a set of genes correlated with the HSF1 expression, indicating a consistent transcriptional program involving HSF1 in different cancer types (Fig. 1b). After multiple testing corrections, we found 114 genes associated with HSF1 expression (*p* < 0.05), and only 17 anti-correlated genes (*p* < 0.05) for all cancer types. Gene ontology analysis revealed that most of the correlated genes were involved in pathways such as ribosomal biogenesis (*p* = 1.18.10^−6^), and non-coding RNA metabolic processes (*p* = 7.49.10^−5^)(Fig. 1c). No significant global enrichment was found for negatively correlated expression. These results support previous works indicating that HSF1 overexpression in cancer is not associated with HSPs expression in cancer, but rather linked to protein translation, and RNA processing processes to support cell proliferation [8, 18, 32].

### HSF1 CNA drives HSF1 expression

**Fig. 2:**
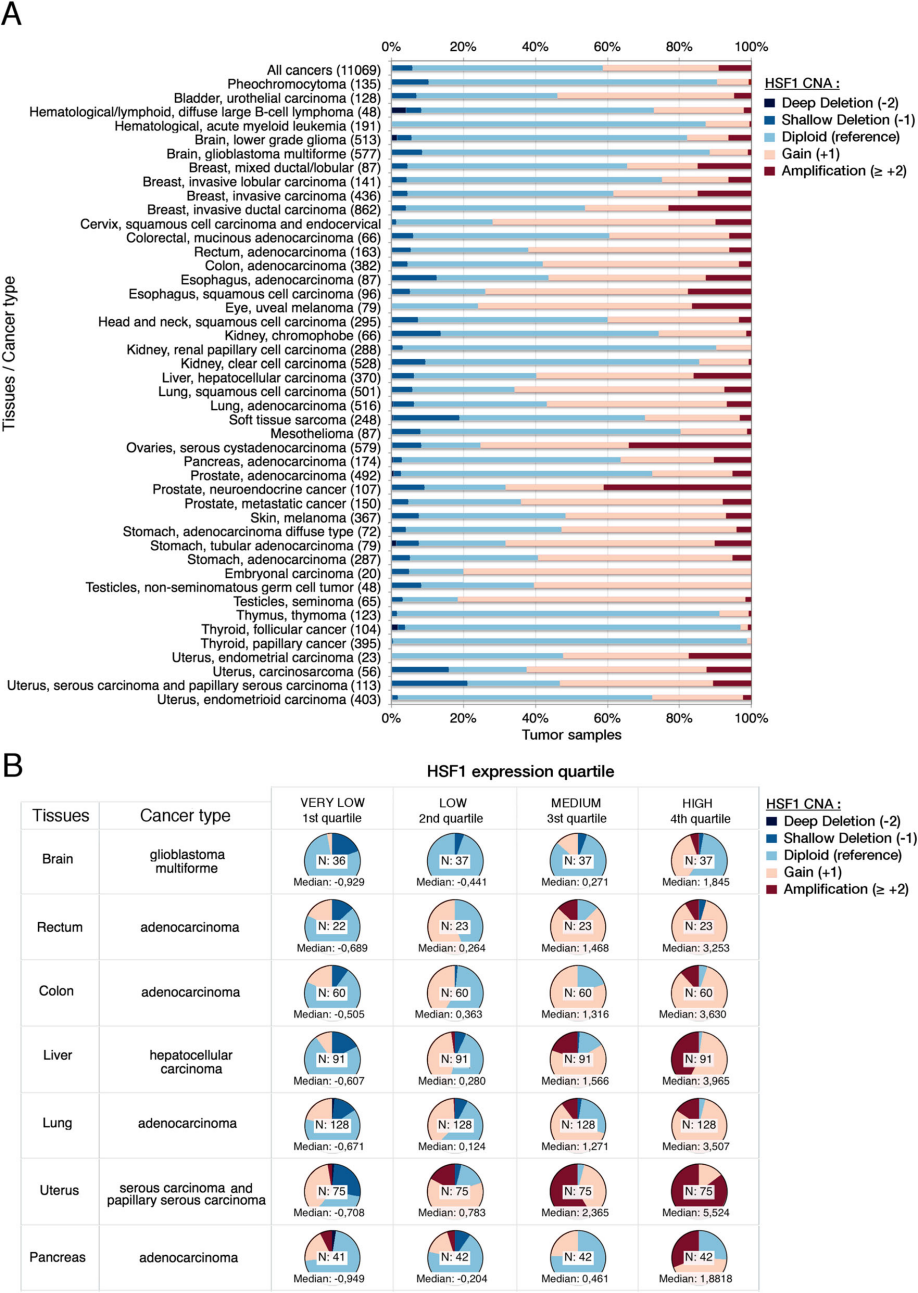
Copy number alteration of HSF1 drives HSF1 expression in cancers. **a** Distribution of HSF1 copy number alteration per different cancer types. Numbers in brackets indicate the number of patient samples per cancer type. **b** Pie charts of HSF1 CNA as a function of the HSF1 quartile expression per histopathological cancer types. Color annotations are described in the legend. Each pie chart displays the number of tumors analyzed and the median value of HSF1 for all samples in the category. Tumors having both CNA data and HSF1 were included. Abbreviations: *N*, number of samples; deep deletion, − 2 copies; shallow deletion, − 1 copy, diploid is the reference with normal HSF1 copy number; gain, + 1 copy; amplification, + 2, or more copies

We first look at the HSF1 locus only, analysis of HSF1 CNA distribution showed that deep deletion and shallow deletion represented a small proportion of tumor, while gain or amplification of HSF1 is often overrepresented (Fig. 2a) in particular for the following histological subtypes: testicular seminoma (82%), uveal melanoma (76%), esophageal squamous cell carcinoma (74%), and neuroendocrine prostate cancer (68%). When assessing the link between CNA and expression for HSF1, we categorized patient samples having both CNA and expression data. In most of the histological subtype, higher copy number of HSF1 tends to overexpress HSF1, whereas the groups having low expression of HSF1 have fewer copies of HSF1 (Fig. 2b, Additional file 1: Appendix 3 displays the complete analysis).

### 8q24.3 CNA drives mainly the expression of HSF1 and 8q24.3 genes

**Fig. 3:**
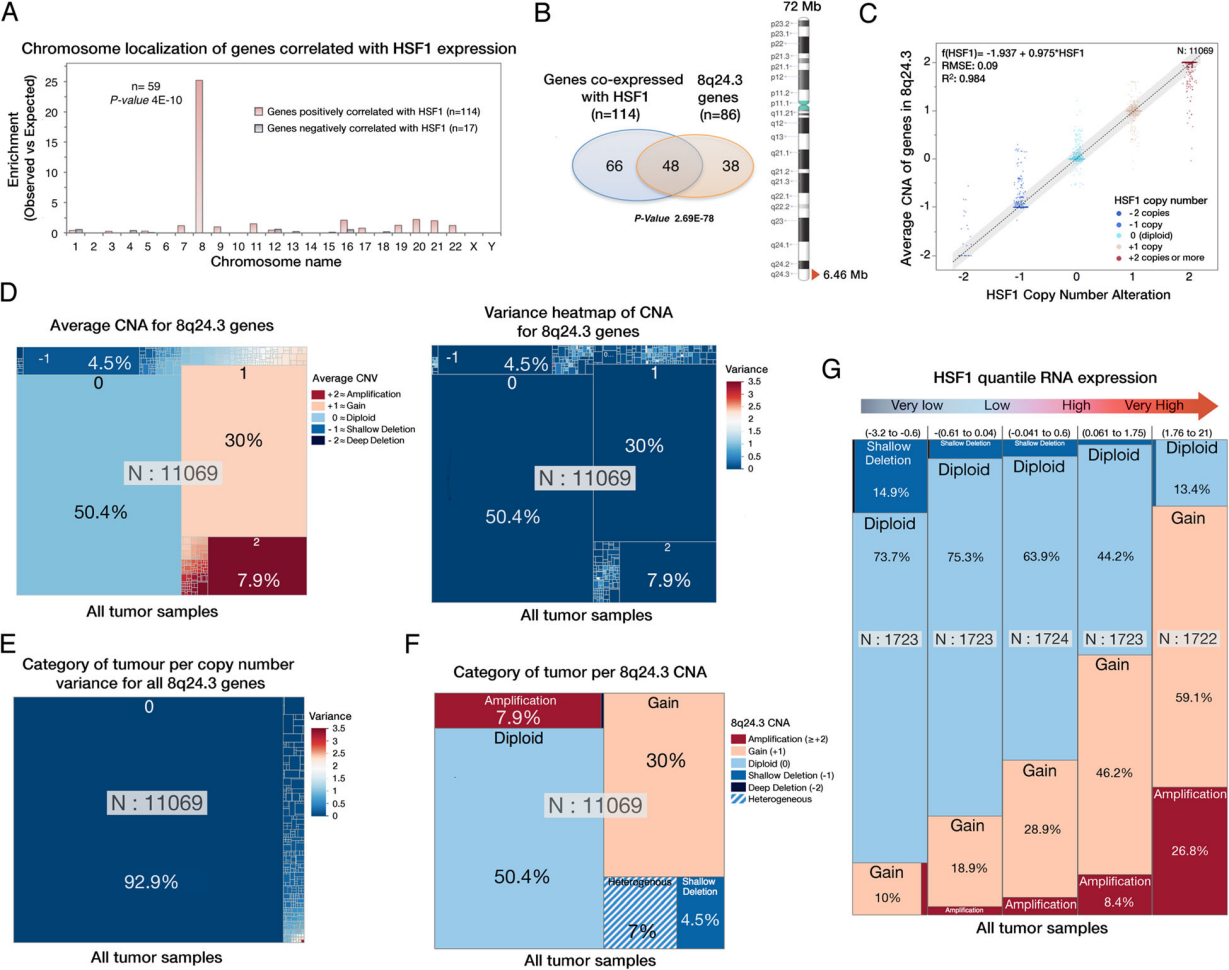
Copy number alteration 8q24.3 is the main inductor for Hsf1 expression. **a** Distribution per chromosome of the positively and negatively correlated expressed genes with HSF1 expression (see Fig. 1c) in all histopathological cancer types. *p* values were calculated using the hypergeometric test. **b** Venn diagram showing the proportion of the co-expressed genes with HSF1 presents in the 8q24.3 cytoband. *p* values were calculated with the hypergeometric test. Abbreviations: n, number of genes. **c** Linear correlation between HSF1 CNA and the average CNA of 8q24.3 genes for all samples. Abbreviations: *N*, number of individual; RMSE, root-mean-square error; *R*^2^ = determination coefficient. **d** Categorical treemap of the average CNA of genes present in 8q24.3 for all tumor samples (left panel). Numbers displayed for the treemap represent the average copy number alteration for all genes located in 8q24.3. “0” is the number of extra copy relative to the diploid reference. The left panel displays for each category presented in the right panel the associated variance of the average CNA. Intermediate colors not presented in the color legend represent the average copy number alteration for all genes with non-null variance. **e** Categorical treemap of the CNA variance for all genes located in 8q24.3 for all individuals. 92.9% of the tumor patients display a homogenous 8q24.3 region independently of the copy number alteration. **f** Categorical treemap summarizing categories of tumor displaying different 8q24.3 CNA. Heterogeneous CNA of genes within the 8q24.3 region have been categorized as heterogeneous. **g** Treemap of all tumor samples grouped per quintile of HSF1 expression strength displaying the proportion of different 8q24.3 CNA category. Tumor samples having heterogeneous 8q24.3 copy number are excluded. Abbreviations: *N*, number of individual per categories

Most genes co-expressed with HSF1 were localized on chromosome 8 (59 of 114 genes, *p =* 4.10^−10^)(Fig. 3a) of which, nearly half of them co-localized with HSF1 in the 8q24.3 region (6.46 megabases)(48 genes, *p* = 4.10^−10^)(Fig. 3b) confirming previous reports [18]. Since the HSF1 gene is located in 8q24.3, we assessed if HSF1 overexpression in cancer could be linked to genome organization rather than a global change in transcriptional programming. The relationship of HSF1 CNA with the average copy number alteration of genes localized in 8q24.3 showed a strong correlation (*R*^2^ = 0.984, *n* = 11,069) indicating that copy number of genes located in 8q24.3 evolves together with HSF1 (Fig. 3c). Overview of the average CNA of 8q24.3 genes suggests that only half of the tumor samples (54.1%) displayed a diploid pattern, whereas less than 5% showed deletions and nearly 40% of the cancer patient has 8q24.3 gain, or amplification (Fig. 3d, left panel). The associated heat map shows that most samples had a null variance for the average copy number alteration of 8q24.3 genes pointing that, independently of their copy number; 8q24.3 region remains homogenous in tumors (Fig. 3d, right panel). In fact, 93% of the 11,069 patient samples showed a null variance of CNA for 8q24.3 genes (Fig. 3e). The variance analysis of CNA for all genes located in 8q24.3 showed that only 7% of the patient samples displayed heterogeneity from this region (Fig. 3f)*.* These results are in agreement with a previous pan-cancer study that did not identify significant CNA peaks between HSF1 and the end of 8q24.3 [33]. Therefore, copy number alterations of 8q24.3 genes, including HSF1, are directly linked to 8q24.3 copy number in tumors.

To assess the influence of homogeneous 8q24.3 copy on HSF1 expression, we excluded patient samples carrying heterogeneous copy number of genes localized in 8q24.3. Not surprisingly, when patient samples were sub-grouped by the strength of HSF1 expression, patient samples overexpressing HSF1 display a higher amount of 8q24.3 copy in their genome (Fig. 3g). Similarly, other genes located within the 8q24.3 region, including cancer-related genes (Additional file 1: Appendix 4), displayed similar trends in different tissues (Additional file 1: Appendixes 5 and 6). Yet, linear regressions analysis of 8q24.3 CNA compared to the expression of genes located in 8q24.3 confirmed that HSF1 expression is one of the most correlated genes with 8q24.3 copy number alteration in different tissues (Additional file 1: Appendix 6). These results indicate that 8q24.3 CNA, not only HSF1, triggers a complex transcriptional change to facilitate cancer development and proliferation.

### Clinical characteristics

Next, we evaluated how 8q24.3 copy number in tumor could affect the clinical prognosis, taking into account confounding and interaction by tumor stage (as assessed by means of the likelihood ratio test). Therefore, we excluded all patients having heterogeneous CNAs within 8q24.3 (*n* = 780) and those with a prior malignancy or incomplete 5-year follow-up information (Additional file 1: Appendix 1). In total, 9568 unique individuals were included, of which 54% were female, 51% were 60 years or older, and 28% were diagnosed between 2011 and 2013, as described in Additional file 1: Table S2. In total, 24 different anatomical locations and 45 different histological subtypes were reported with breast (13%), and brain tumors (11%) being most common. Tumors were in situ or stage 0–1 in 18%, stage 2 in 11%, stage 3 in 15% and stage 4 in 7%, and information was missing in 50%.

In total, 5174 (54%) of cancers were diploid for 8q24.3 (Additional file 1: Table S2), 12 (0.1%) had deep deletion, 454 (5%) shallow deletion, and respectively 3082 (32%) and 9568 (9%) showed gain or amplification. Women presented more frequently with diploidy and amplification (55% and 11%) than men (53% and 7%)(*p* < 0.0001), and the proportion of diploidy decreased by age (65% in < 40 years, 50% in ≥70 years; *p* < 0.0001). Diploidy was most common in thyroid cancers (97%), thymus cancer (89%), and hematological cancer (83%). The 8q24.3 gain was especially common in testicular cancer (75%) and head-and-neck cancer (62%). Diploidy was more common in stage 0–1 or in situ tumors (59%) compared to stage 4 (42%)(*p* < 0.0001).

### Overall prognosis

At 5 years after diagnosis, 28% has died, 47% were alive without recurrence, and 11% had a recurrence but were still alive. Recurrence information was missing in 19% of individuals who survived. Of those who died, 49% presented with 8q24.3 diploidy, of those who were alive, and disease-free, 59% (*p* < 0.00001, Additional file 1: Table S2).

**Fig. 4:**
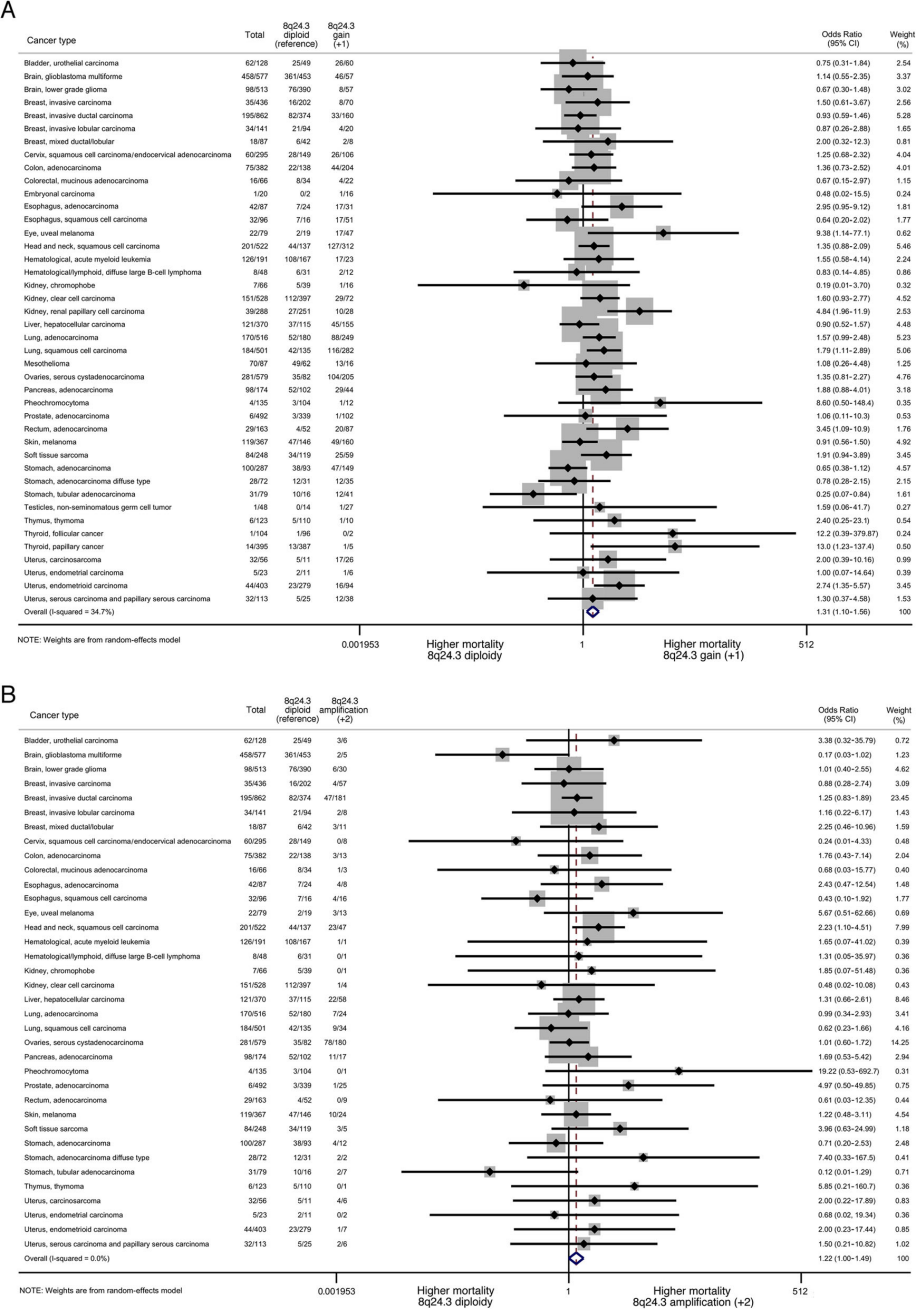
Forest plots assessing the association between 8q24.3 gain (**a**) and amplification (**b**) and 5-year mortality per histopathological subtype of cancer using diploidy as a reference. The numbers in the columns refer to the total number of individuals presenting with each cancer type, and the number who died within 5 years, for the total group of individuals, those with diploidy (reference) and those with gain (**a**) or amplification (**b**). Individuals with deletion are not included in these analyses, since diploidy is considered the reference. Weights are derived from a random-effects model. Abbreviations: OR, odds ratio; CI, confidence interval

The two-step meta-analyses, weighted by study using 8q24.3 diploidy as reference, showed a 32% (OR = 1.32, [95% CI 1.03–1.69]), 36% (1.36, [1.15–1.60]), and 23% (1.23, [1.01–1.51]) increased 5-year mortality for shallow/deep deletion, gain, and amplification, respectively (all low heterogeneity), with similar results when weighted by anatomical location (moderate heterogeneity) or histological subtype (low heterogeneity) (Additional file 1: Table S3). The forest plots for gain and amplification by histological subtype are presented in Fig. 4. The odds of disease-free survival were 20–25% lower in those without 8q24.3 diploidy in all models (Additional file 1: Table S3).

The one-step meta-analysis approach was used to assess if the effects of 8q24.3 ploidy on mortality remained after adjustment for confounding and interaction using diploidy as a reference. The unadjusted 5-year mortality (model 1, *n* = 9568) showed similar results as above, with respectively 42%, 31%, and 20% increased risks for shallow/deep deletion, gain, and amplification (Table 1). After adjustments for age, sex, calendar period, and clustering by study (model 2, *n* = 7593), the results remained stable yet lost significance. Since interaction between 8q24.3 ploidy and tumor stage was present (*p* = 0.0016) but not between ploidy and HSF1 expression (*p* = 0.0976) (data not shown), model 3 (*n* = 4110) is additionally adjusted for interaction with tumor stage and confounding by HSF1-expression, resulting in doubled risks among those with gain (OR = 1.98, [1.22–3.21]) or amplification (OR = 2.19, [1.13–4.26])(Table 1).

**Table 1:** The risk of 5-year mortality by 8q24.3 copy number alteration—categorized by anatomical location of each cancer—and expressed as odds ratios [OR] and 95% confidence intervals [CI]

	Total			Model 1*(*n* = 9568)			Model 2** (*n* = 7593)			Model 3*** (*n* = 4110)		
	% died	% diploid	n	Deletions (− 1 or − 2)	Gain (+ 1)	Amplification (≥ + 2)	Deletions (− 1 or − 2)	Gain (+ 1)	Amplification (≥ + 2)	Deletions (− 1 or − 2)	Gain (+ 1)	Amplification (≥ + 2)
Adrenal glands	1.8	79.8	109		8.60 [0.5–148.39]							
Bladder	44.8	39.1	87		0.75 [0.31–1.84]							
Blood	56.3	83.2	238		0.87 [0.42–1.80]							
Brain	51.4	80.2	1,02	1.65 [0.94–2.90]	0.84 [0.56–1.24]	0.27 [0.12–0.60]	1.23 [0.79–1.92]	0.83 [0.43–1.59]	0.68 [0.37–1.25]			
Breast	18.6	56.0	1272	1.17 [0.55–2.50]	1.05 [0.72–1.52]	1.31 [0.92–1.86]						
Cervical	19.9	52.5	276	0.78 [0.21–2.88]	1.25 [0.68–2.32]							
Colorectal	18.9	38.0	513	3.64 [1.31–10.17]	1.51 [0.93–2.46]	0.90 [0.25–3.25]	4.12 [1.15–14.82]	1.75 [1.32–2.31]	0.78 [0.23–2.64]			
Esophagus	38.4	25.2	159	1.16 [0.32–4.23]	1.32 [0.60–2.88]	0.93 [0.32–2.70]						
Eyes	26.7	24.0	75		9.38 [1.14–77.14]	5.67 [0.51–62.66]						
Head and neck	39.1	27.8	468		1.35 [0.88–2.09]	2.23 [1.10–4.51]		1.40 [0.75–2.62]	2.68 [2.17–3.30]			
Kidney	23.0	77.6	738	0.49 [0.22–1.11]	1.83 [1.17–2.87]		0.41 [0.21–0.82]	1.70 [0.73–3.96]		0.52 [0.31–0.85]	0.60 [0.23–1.62]	
Liver	32.6	31.9	313	1.49 [0.52–4.26]	0.90 [0.52–1.57]	1.31 [0.66–2.61]						
Lung	33.7	32.8	811	1.49 [0.76–2.90]	1.70 [1.22–2.36]	0.76 [0.37–1.56]	1.97 [1.02–3.80]	1.76 [1.24–2.51]	0.80 [0.44–1.46]	1.24 [0.34–4.51]	1.77 [1.06–2.97]	0.40 [0.10–1.65]
Mesenchyme	33.5	55.1	185	1.47 [0.60–3.56]	1.91 [0.94–3.89]							
Mesothelium	81.3	73.3	75		1.08 [0.26–4.48]							
Ovarian	47.5	16.1	503	1.81 [0.83–3.94]	1.35 [0.81–2.27]	1.01 [0.60–1.72]	1.83 [1.39–2.41]	1.53 [1.15–2.05]	1.33 [0.91–1.93]	1.52 [1.26–1.83]	7.24 [6.44–8.13]	9.73 [7.19–13.16]
Pancreas	55.8	60.4	154		1.87 [0.88–4.01]	1.69 [0.53–5.42]						
Prostate	0.9	57.7	647	5.61 [0.56–56.12]	0.68 [0.07–6.56]	1.84 [0.19–17.96]	24.75 [0.8–765]	1.12 [0.76–1.64]	4.41 [3.41–5.71]			
Skin	31.6	42.1	335	1.22 [0.48–3.11]	0.91 [0.56–1.50]	1.22 [0.48–3.11]						
Stomach	36.3	34.8	397	0.91 [0.33–2.53]	0.60 [0.39–0.93]	0.70 [0.26–1.86]	0.93 [0.13–6.52]	0.60 [0.37–0.98]	0.56 [0.36–0.87]			
Testicle	1.6	19.2	125									
Thymus	5.3	89.4	113									
Thyroid	2.4	97.4	464									
Uterus	18.1	60.7	491	4.31 [1.70–10.88]	3.53 [2.11–5.90]	4.31 [1.62–11.48]	4.84 [2.75–8.51]	3.67 [2.03–6.66]	4.58 [1.43–14.6]	1.13 [0.16–7.78]	1.99 [1.28–3.08]	2.08 [0.06–75.09]
Total	28.3	54.1	9568	1.42 [1.16–1.73]	1.31 [1.19–1.45]	1.20 [1.02–1.40]	1.37 [0.88–2.15]	1.31 [0.78–2.20]	1.41 [0.76–2.61]	1.23 [0.69–2.16]	1.98 [1.22–3.21]	2.19 [1.13–4.26]

*Model 1 is unadjusted for any confounders

**Model 2 is adjusted for age, sex, calendar period, and clustering by study

***Model 3 is additionally adjusted for interaction with tumor stage and confounding by HSF1

### Prognosis per anatomical location

The two-step meta-analysis approach (Fig. 4) shows that, compared to diploidy as reference, gain was associated with a significantly increased mortality for 7 subtypes, including papillary thyroid cancer (OR = 13.00), uveal melanoma (OR = 9.38), and renal papillary cell carcinoma (OR = 4.84); and a decreased mortality for tubular gastric adenocarcinoma (OR = 0.25). For amplification, mortality was significantly higher than diploidy for squamous cell head and neck carcinoma (OR = 2.23). For each anatomical location, all three models were presented if feasible (Table 1). For deletion, model 2 showed a significantly increased 5-year mortality for cancer of the uterus (OR = 4.84), colorectal (OR = 4.12), lung (OR = 1.91), and ovaries (OR = 1.83), and decreased risk of kidney cancer (OR = 0.41). After full adjustment (model 3), only the results for ovarian (OR = 1.52) and kidney cancer (OR = 0.52) were confirmed. For gain, model 2 found significant associations for cancer of the uterus (OR = 3.67), lung (OR = 1.76), colorectal (OR = 1.75), ovaries (OR = 1.53), and stomach (OR = 0.60), which were confirmed in model 3 for cancer of the uterus (OR = 1.99), lung (1.77), and ovaries (OR = 7.24). Amplification was associated with cancer of the uterus (OR = 4.58), prostate (OR = 4.41), head and neck (OR = 2.68), and stomach (OR = 0.56) in model 2, and ovaries in model 3 (OR = 9.73).

### Prognosis per histological subtype

The 5-year mortality (model 2) was significantly higher for 8q24.3 deletion in serous cystadenocarcinoma of the ovaries (OR = 1.83) and squamous cell carcinoma of the lungs (OR = 1.79); and for gain/amplification in endometrial carcinoma (OR = 3.63), rectal adenocarcinoma (OR = 2.43), prostate adenocarcinoma (OR = 1.92), squamous cell carcinoma of the lungs (OR = 1.92), serous cystadenocarcinoma (OR = 1.44), chromophobe renal cell carcinoma (OR = 1.38), and tubular adenocarcinoma of the stomach (OR = 0.21)(Additional file 1: Tables S4-S5).

## Discussion

Here, we showed that expression of HSF1 as well as other genes localized in 8q24.3 are tightly linked to 8q24.3 copy number. This large individual patient data meta-analysis approach showed evidence for higher 5-year mortality among individuals with 8q24.3 deletions, gain, and amplification. These overall results remained rather stable after adjustment for confounders and interaction by tumor stage, which supports a causal relationship that cannot be explained by tumor stage, HSF1 expression, or by the assessed confounders. Up to 9-fold increased risks were found for specific cancer types. This supports a potential causal relationship between 8q24.3 CNA and prognosis at least in some histological subtypes—although protective effects were found in a limited number of cancer types (kidney and stomach).

Therefore, this suggests that 8q24.3 CNA and its complex transcriptional change imply either responsive or resistance in treatment, which needs further clinical and molecular investigations. For example in the different histological subtypes, it would be interesting to investigate with other known genomic biomarkers important in cancer as well exploring the link with complex karyotypes to explore assess the link with the stress of protein unbalances in tumors. It is also worthwhile to understand why both a deletion and a gain of 8q24.3 can lead to a poor prognosis in some tissues such as lung, colorectal, and ovaries. Possibly copy number change in 8q24.3 could alter transcriptional programming or could be associated with other genomic change including translocations and inversions that alters the resistance to treatment or favorize tumor growth.

The main strength of this meta-analysis is that the results are based on a large population with available data on an individual level. Both applied meta-analyses approaches obtained similar results, with low to moderate statistical heterogeneity for all analyses. Yet, information was incomplete or missing for important prognostic variables such as tumor stage (missing in 50%) and confounders such as body mass index, smoking, and alcohol intake. Therefore, the most adjusted models were conducted on 42% of the cohort (complete case analysis), resulting in reduced power, which in turn contributed to the loss of statistical significance compared to the unadjusted analyses. Residual confounding cannot be ruled out. Consequently, the results have to be interpreted with caution, in particular, for specific histological subtypes with the low number of patients included in the analyses.

Our findings may have substantial implications for the understanding and interpretation of biomarkers in cancer research and clinical investigations. Indeed, no less than 5000 publications were found for 16 popular genes including HSF1 in 8q24.3, of which 800 publications related to cancer field (Additional file 1: Appendix 4), mainly because those genes were found overexpressed in cancer (Additional file 1: Appendix 5).

## Conclusions

Integration of 8q24.3 CNA detection may have substantial implications for interpreting the molecular pathogenesis of cancer. In a general aspect, our work indicates that histological diagnoses using biomarkers can be tightly linked to large CNA associated with complex gene expression pattern, pointing out the importance of understanding molecular pathogenesis to optimize cancer treatment.

## Supplementary information

**Additional file 1 **: Table S1. Data Collected. Table S2. Descriptive characteristics of all individuals (*n* = 9568) with 8q24.3 homogenous copy number alteration information, first cancer diagnosis and 5-year follow-up. Table S3. Results of the 2-step individual patient data meta-analyses to assess the effect of 8q24.3 copy number variants on prognosis. Table S4. Five-year mortality by 8q24.3 copy number alteration using diploidy as a reference. Table S5. Five-year mortality of 15 cancer types with most cases of 8q24.3 gain and amplification using diploidy as a reference. Appendix 1. Flow chart illustrating selection of individuals for individual patient data meta-analysis. Appendix 2. Overview of HSF1 expression per histological subtype. Appendix 3. Expression of HSF1 in function of HSF1 copy number alteration per histological subtype. Appendix 4. Number of publication and description of selected cancer-related genes per field located in 8q24.3 cytoband. Appendix 5. Influence of 8q24.3 copy number alteration on other cancer-related genes expression located in 8q24.3 loci and comparison of expression with HSF1 per tissue. Appendix 6. Simple linear regressions of predicted 8q24.3 copy number alteration compared to the expression of genes located in 8q24.3 per tissue.

## Data Availability

The published results showed available form authors here are in whole or part based upon data generated by the TCGA Research Network (http://cancergenome.nih.gov/) and its TCGA Pan-Cancer Analysis Working Group and obtained from the cBioportal for Cancer Genomics [25, 26].
