# Supplementary material for: Copy number of 8q24.3 drives HSF1 expression and patient outcome in cancer: an individual patient data meta-analysis
Source: Hum Genomics. 2019 Nov 7;13:54. doi: 10.1186/s40246-019-0241-3 (PMC6836670; doi:10.1186/s40246-019-0241-3)
Supplement: Supplementary file 1 — Additional file 1 : Table S1. Data Collected. Table S2. Descriptive characteristics of all individuals (n = 9568) with 8q24.3 homogenous copy number alteration information, first cancer diagnosis and 5-year follow-up. Table S3. Results of the 2-step individual patient data meta-analyses to assess the effect of 8q24.3 copy number variants on prognosis. Table S4. Five-year mortality by 8q24.3 copy number alteration using diploidy as a reference. Table S5. Five-year mortality of 15 cancer types with most cases of 8q24.3 gain and amplification using diploidy as a reference. Appendix 1. Flow chart illustrating selection of individuals for individual patient data meta-analysis. Appendix 2. Overview of HSF1 expression per histological subtype. Appendix 3. Expression of HSF1 in function of HSF1 copy number alteration per histological subtype. Appendix 4. Number of publication and description of selected cancer-related genes per field located in 8q24.3 cytoband. Appendix 5. Influence of 8q24.3 copy number alteration on other cancer-related genes expression located in 8q24.3 loci and comparison of expression with HSF1 per tissue. Appendix 6. Simple linear regressions of predicted 8q24.3 copy number alteration compared to the expression of genes located in 8q24.3 per tissue. [file 40246_2019_241_MOESM1_ESM.pdf]

DATA SUPPLEMENT

Table of contents

Table S1 Data Collected .... Page 2

Table S2 Descriptive Characteristics of All Individuals (n=9,568) with 8q24.3 Homogenous Copy Number Alteration Information, First Cancer Diagnosis and 5-year Follow-up.. Page 3-4

Table S3 Results of the 2-step Individual Patient Data Meta-Analyses to Assess the Effect of 8q24.3 Copy Number Variants on Prognosis.. Page 5

Table S4 Five-year Mortality by 8q24.3 Copy Number Alteration using Diploidy as a Reference .... Page 6

Table S5 Five-year Mortality of 15 cancer types with Most Cases of 8q24.3 Gain and Amplification using Diploidy as a Reference .... Page 7

Appendix 1 Flow chart Illustrating Selection of Individuals for Individual Patient Data Meta-Analysis .... Page 8

Appendix 2 Overview of HSF1 Expression per Histological Subtype.............................. Page 9

Appendix 3 Expression of HSF1 in Function of HSF1 Copy Number Alteration per Histological subtype .... Page 10

Appendix 4 Number of Publication and Description of Selected Cancer-Related Genes per Field Located in 8q24.3 cytoband.. Page 11-12

Appendix 5 Influence of 8q24.3 Copy Number Alteration on Other Cancer-Related Genes Expression Located in 8q24.3 Loci and Comparison with HSF1 Related Expression per Tissue .... Page 13-24

Appendix 6. Simple Linear Regressions of Predicted 8q24.3 Copy Number Alteration Compared to the Expression of Genes Located in 8q24.3 per Tissue .... Page 25

Table S1

Cancer type	Total number of patient	Morbidity data	Disease Free data	CNA data	Expression data
Hematological, acute myeloid leukemia	191	190	187	191	166
Bladder, urothelial carcinoma	128	128	100	122	126
Brain, lower grade glioma	513	510	474	494	513
Breast, invasive ductal carcinoma	862	862	..	737	862
Breast, invasive lobular carcinoma	141	141	..	127	141
Breast, mixed ductal/lobular	87	87	..	65	87
Head and neck, squamous cell carcinoma	295	293	255	283	292
Colon, adenocarcinoma	382	381	330	367	240
Skin, melanoma	367	357	314	354	367
Stomach, adenocarcinoma diffuse type	72	72	58	71	49
Embryonal carcinoma	20	20	19	19	20
Uterus, endometrial carcinoma	23	22	21	19	13
Oesophagus, adenocarcinoma	87	87	65	71	87
Oesophagus, squamous cell carcinoma	96	96	76	88	95
Thyroid, follicular cancer	104	104	103	102	104
Brain, glioblastoma multiforme	577	569	417	556	147
Cervix, squamous cell carcinoma and endocervical adenocarcinoma	522	519	392	500	514
Breast, invasive carcinoma	436	243	..	343	173
Kidney, chromophobe	66	64	62	65	66
Kidney, clear cell carcinoma	528	526	429	523	525
Kidney, renal papillary cell carcinoma	288	285	266	287	288
Liver, hepatocellular carcinoma	370	367	317	347	364
Lung, adenocarcinoma	516	505	430	481	512
Lung, squamous cell carcinoma	501	493	372	474	498
Hematological/lymphoid, diffuse large B-cell lymphoma	48	46	42	47	48
Mesothelioma	87	86	..	83	87
Prostate, metastatic cancer	150	..	..	117	118
Colorectal, mucinous adenocarcinoma	66	66	56	63	23
Prostate, neuroendocrine cancer	107	..	..	82	46
Testicles, non-seminomatous germ cell tumor	48	48	47	44	48
Ovaries, serous cystadenocarcinoma	579	563	481	505	300
Pancreas, adenocarcinoma	174	173	130	168	167
Thyroid, papillary cancer	395	393	380	394	393
Pheochromocytoma	135	135	..	129	135
Prostate, adenocarcinoma	492	491	485	477	491
Rectum, adenocarcinoma	163	161	145	156	91
Testicles, seminoma	65	65	65	64	65
Soft tissue sarcoma	248	247	218	219	246
Stomach, adenocarcinoma	287	280	218	266	258
Thymus, thymoma	123	121	117	123	119
Stomach, tubular adenocarcinoma	79	79	61	68	34
Uterus, carcinosarcoma	56	56	56	51	56
Uterus, endometrioid carcinoma	403	402	378	384	105
Uterus, serous carcinoma and papillary serous carcinoma	113	113	98	84	58
Eye, uveal melanoma	79	67	59	79	79
**All cancers**	**11,069**	**10,513**	**7,723**	**10,289**	**9,216**

Table S1. Data Collected from cBioportal.
Data collected from cBioportal portal which includes peer-reviewed studies, METABRIC data (Molecular Taxonomy of Breast Cancer International Consortium) and unpublished data from The Cancer Genome Atlas (TCGA). The reported numbers represent the number of patients with available data Abbreviations: CNA : Copy Number Alteration; (..) : Non available

Table S2 (1 of 2)

	Deep deletion (-2)		Shallow deletion (-1)		Diploid (reference)		Gain (+1)		Amplification (+2)		Total
	N	%	N	%	N	%	N	%	N	%	N
**Total**	12	0·1	454	4·7	5.174	54·1	3.082	32·2	846	8·8	9.568
**Sex**											
Female	5	0·1	246	4·8	2.838	55·4	1.478	28·8	558	10·9	5.125
Male	7	0·2	204	4·6	2.315	52·5	1.599	36·3	285	6·5	4,410
Missing	0	0·0	4	12·1	21	63·6	5	15·2	3	9·1	33
**Age, years**											
<40	2	0·2	38	3·9	632	64·5	252	25·7	56	5·7	980
40-49	1	0·1	54	4·4	725	58·6	338	27·3	119	9·6	1.237
50-59	3	0·1	89	4·1	1.214	55·4	688	31·4	197	9·0	2.191
60-69	4	0·2	125	4·9	1.34	52·1	878	34·2	224	8·7	2.571
70-95	2	0·1	122	5·4	1.13	49·8	848	37·4	168	7·4	2.27
Missing	0	0·0	26	8·2	133	41·7	78	24·5	82	25·7	319
**HSF1-expression (quartiles)**											
-3·17 to -0·45	9	0·5	245	12·5	1.481	75·4	209	10·6	20	1·0	1.964
-0·45 to 0·27	1	0·0	82	4·0	1.431	70·7	463	22·9	48	2·4	2.025
0·27 to 1·39	0	0·0	34	1·7	1.063	53·4	780	39·2	113	5·7	1,990
1·39 to 21·06	0	0·0	14	0·7	325	16·2	1.169	58·2	500	24·9	2.008
Missing	2	0·1	79	5·0	874	55·3	461	29·2	165	10·4	1.581
**Tumour stage**											
Stage 0-1 or in situ	1	0·1	79	4·6	1.024	59·2	550	31·8	75	4·3	1.729
Stage 2	2	0·2	46	4·6	444	44·2	436	43·4	76	7·6	1.004
Stage 3	3	0·2	89	6·2	556	38·6	614	42·7	177	12·3	1.439
Stage 4	1	0·2	22	3·5	265	41·6	273	42·9	76	11·9	637
Missing	5	0·1	218	4·6	2.885	60·6	1.209	25·4	442	9·3	4.759
**Year of diagnosis**											
1978-2005	2	0·1	112	6·2	923	50·8	622	34·2	159	8·7	1.818
2006-2008	2	0·1	74	4·9	803	52·8	529	34·8	112	7·4	1.52
2009-2010	2	0·1	69	4·4	888	56·2	535	33·9	85	5·4	1.579
2011-2013	5	0·2	117	4·3	1.511	55·8	922	34·1	151	5·6	2.706
Missing	1	0·1	82	4·2	1.049	53·9	474	24·4	339	17·4	1.945
**5-years outcome**											
Alive and disease free	8	0·2	193	4·3	2.629	58·9	1.41	31·6	225	5·0	4.465
Recurred/Progressed	3	0·3	56	5·4	499	47·8	373	35·8	112	10·7	1.043
Died	1	0·0	153	5·6	1.338	49·4	968	35·7	249	9·2	2.709
Recurrence not reported	0	0·0	52	3·8	708	52·4	331	24·5	260	19·2	1.351
**Anatomical location**											
Adrenal glands	0	0·0	10	9·2	87	79·8	11	10·1	1	0·9	109
Bladder	0	0·0	4	4·6	34	39·1	45	51·7	4	4·6	87
Blood	1	0·4	2	0·8	198	83·2	35	14·7	2	0·8	238
Brain	2	0·2	54	5·3	818	80·2	111	10·9	35	3·4	1,020
Breast	0	0·0	45	3·5	712	56·0	258	20·3	257	20·2	1.272
Cervix	0	0·0	19	6·9	145	52·5	104	37·7	8	2·9	276
Colorectal	0	0·0	18	3·5	195	38·0	278	54·2	22	4·3	513
Esophagus	0	0·0	13	8·2	40	25·2	82	51·6	24	15·1	159
Eyes	0	0·0	0	0·0	18	24·0	45	60·0	12	16·0	75
Head and Neck	0	0·0	4	0·9	130	27·8	292	62·4	42	9·0	468
Kidney	0	0·0	57	7·7	573	77·6	105	14·2	3	0·4	738
Liver	0	0·0	17	5·4	100	31·9	141	45·0	55	17·6	313
Lung	0	0·0	45	5·5	266	32·8	450	55·5	50	6·2	811
Mesenchyme	0	0·0	28	15·1	102	55·1	50	27·0	5	2·7	185
Mesothelium	0	0·0	4	5·3	55	73·3	16	21·3	0	0·0	75
Ovaries	2	0·4	36	7·2	81	16·1	205	40·8	179	35·6	503
Pancreas	1	0·6	4	2·6	93	60·4	42	27·3	14	9·1	154
Prostate	3	0·5	20	3·1	373	57·7	183	28·3	68	10·5	647
Skin	0	0·0	22	6·6	141	42·1	150	44·8	22	6·6	335
Stomach	0	0·0	17	4·3	138	34·8	222	55·9	20	5·0	397
Testicles	0	0·0	6	4·8	24	19·2	94	75·2	1	0·8	125
Thymus	0	0·0	2	1·8	101	89·4	9	8·0	1	0·9	113
Thyroid	2	0·4	4	0·9	452	97·4	6	1·3	0	0·0	464
Uterus	1	0·2	23	4·7	298	60·7	148	30·1	21	4·3	491

Table S2. Descriptive Characteristics of All Individuals (n=9,568) with homogenous 8q24.3 Copy Number Alteration Information, First Cancer Diagnosis and Five-year Follow-Up.

Table S2 (1 of 2)

	Deep deletion (-2)		Shallow deletion (-1)		Diploid (reference)		Gain (+1)		Amplification (+2)		Total
	N	%	N	%	N	%	N	%	N	%	N
**Total**	12	0.1	454	4.7	5,174	54.1	3,082	32.2	846	8.8	9,568
**Histological subtype**											
Bladder, urothelial carcinoma	0	0.0	4	4.6	34	39.1	45	51.7	4	4.6	87
Brain, glioblastoma multiforme	0	0.0	39	7.2	441	81.7	55	10.2	5	0.9	540
Brain, lower grade glioma	2	0.4	15	3.1	377	78.5	56	11.7	30	6.3	480
Breast, invasive carcinoma	0	0.0	14	4.1	202	58.9	70	20.4	57	16.6	343
Breast, invasive ductal carcinoma	0	0.0	22	3.0	374	50.7	160	21.7	181	24.6	737
Breast, invasive lobular carcinoma	0	0.0	5	3.9	94	74.0	20	15.7	8	6.3	127
Breast, mixed ductal/lobular	0	0.0	4	6.2	42	64.6	8	12.3	11	16.9	65
Cervix, squamous cell carcinoma and endocervical adenocarcinoma	0	0.0	19	6.9	145	52.5	104	37.7	8	2.9	276
Colon, adenocarcinoma	0	0.0	8	2.5	113	36.0	181	57.6	12	3.8	314
Colorectal, mucinous adenocarcinoma	0	0.0	4	7.0	32	56.1	19	33.3	2	3.5	57
Embryonal carcinoma	0	0.0	1	5.3	2	10.5	16	84.2	0	0.0	19
Esophagus, adenocarcinoma	0	0.0	8	11.3	24	33.8	31	43.7	8	11.3	71
Esophagus, squamous cell carcinoma	0	0.0	5	5.7	16	18.2	51	58.0	16	18.2	88
Eye, uveal melanoma	0	0.0	0	0.0	18	24.0	45	60.0	12	16.0	75
Head and neck, squamous cell carcinoma	0	0.0	4	0.9	130	27.8	292	62.4	42	9.0	468
Haematological, acute myeloid leukaemia	0	0.0	0	0.0	167	87.4	23	12.0	1	0.5	191
Haematological/lymphoid, diffuse large B-cell lymphoma	1	2.1	2	4.3	31	66.0	12	25.5	1	2.1	47
Kidney, chromophobe renal cell carcinoma	0	0.0	8	13.8	35	60.3	14	24.1	1	1.7	58
Kidney, clear cell carcinoma	0	0.0	43	9.6	337	75.4	65	14.5	2	0.4	447
Kidney, renal papillary cell carcinoma	0	0.0	6	2.6	201	86.3	26	11.2	0	0.0	233
Liver, hepatocellular carcinoma	0	0.0	17	5.4	100	31.9	141	45.0	55	17.6	313
Lung, adenocarcinoma	0	0.0	25	6.3	151	37.8	205	51.2	19	4.8	400
Lung, squamous cell carcinoma	0	0.0	20	4.9	115	28.0	245	59.6	31	7.5	411
Mesothelioma	0	0.0	4	5.3	55	73.3	16	21.3	0	0.0	75
Ovaries, serous cystadenocarcinoma	2	0.4	36	7.2	81	16.1	205	40.8	179	35.6	503
Pancreas, adenocarcinoma	1	0.6	4	2.6	93	60.4	42	27.3	14	9.1	154
Pheochromocytoma	0	0.0	10	9.2	87	79.8	11	10.1	1	0.9	109
Prostate, adenocarcinoma	3	0.7	8	1.8	316	70.5	99	22.1	22	4.9	448
Prostate, metastatic cancer	0	0.0	5	4.3	38	32.5	65	55.6	9	7.7	117
Prostate, neuroendocrine cancer	0	0.0	7	8.5	19	23.2	19	23.2	37	45.1	82
Rectum, adenocarcinoma	0	0.0	6	4.2	50	35.2	78	54.9	8	5.6	142
Skin, melanoma	0	0.0	22	6.6	141	42.1	150	44.8	22	6.6	335
Soft tissue sarcoma	0	0.0	28	15.1	102	55.1	50	27.0	5	2.7	185
Stomach, adenocarcinoma	0	0.0	11	4.2	92	35.2	146	55.9	12	4.6	261
Stomach, adenocarcinoma diffuse type	0	0.0	2	2.9	30	43.5	35	50.7	2	2.9	69
Stomach, tubular adenocarcinoma	0	0.0	4	6.0	16	23.9	41	61.2	6	9.0	67
Testicles, non-seminomatous germ cell tumor	0	0.0	3	7.1	13	31.0	26	61.9	0	0.0	42
Testicles, seminoma	0	0.0	2	3.1	9	14.1	52	81.3	1	1.6	64
Thymus, thymoma	0	0.0	2	1.8	101	89.4	9	8.0	1	0.9	113
Thyroid, follicular cancer	2	2.0	2	2.0	92	93.9	2	2.0	0	0.0	98
Thyroid, papillary cancer	0	0.0	2	0.5	360	98.4	4	1.1	0	0.0	366
Uterus, carcinosarcoma	0	0.0	7	15.6	8	17.8	24	53.3	6	13.3	45
Uterus, endometrial carcinoma	0	0.0	0	0.0	10	58.8	5	29.4	2	11.8	17
Uterus, endometrioid carcinoma	1	0.3	3	0.8	260	72.8	86	24.1	7	2.0	357
Uterus, serous carcinoma and papillary serous carcinoma	0	0.0	13	18.1	20	27.8	33	45.8	6	8.3	72

Table S2. Descriptive Characteristics of All Individuals (n=9,568) with homogenous 8q24.3 Copy Number Alteration Information, First Cancer Diagnosis and Five-year Follow-Up.

	Weighted by anatomical location		Weighted by histological subtype		Weighted by study	
	OR [95% CI)	I-squared (%)	OR (95% CI)	I-squared (%)	OR (95% CI)	I-squared (%)
**5-year mortality**						
Diploidy	1.00 (reference)	-	1.00 (reference)	-	1.00 (reference)	-
Shallow or deep deletion (-1 or -2)	1.40 [1.07-1.84]	19.3	1.32 [1.04-1.67]	0.0	1.32 [1.03-1.69]	4.5
Gain (+1)	1.32 [1.07-1.62]	57.7	1.31 [1.10-1.56]	34.7	1.36 [1.15-1.60]	32.6
Amplification (+2)	1.22 [0.89-1.68]	44.0	1.22 [1.00-1.49]	0.0	1.23 [1.01-1.51]	0.0
Gain (+1) or amplification (+2)	1.30 [1.05-1.60]	62.4	1.29 [1.10-1.52]	0.0	1.33 [1.14-1.55]	29.8
Deletion or gain/amplification	1.27 [1.06-1.52]	54.9	1.26 [1.09-1.45]	26.8	1.29 [1.12-1.49]	25.9
**5-year healthy survival**						
Diploidy	1.00 (reference)	-	1.00 (reference)	-	1.00 (reference)	-
Shallow or deep deletion (-1 or -2)	0.73 [0.53-1.02]	38.7	0.78 [0.61-1.01]	4.9	0.77 [0.59-0.99]	7.6
Gain (+1)	0.78 [0.64-0.95]	56.4	0.78 [0.66-0.93]	36.5	0.76 [0.64-0.90]	37.0
Amplification (+2)	0.77 [0.57-1.05]	32.3	0.77 [0.61-0.96]	0.0	0.76 [0.61-0.96]	0.0
Gain (+1) or amplification (+2)	0.79 [0.64-0.97]	62.9	0.78 [0.66-0.92]	37.2	0.76 [0.64-0.90]	39.0
Deletion or gain/amplification	0.80 [0.66-0.97]	61.1	0.79 [0.68-0.92]	32.1	0.77 [0.66-0.90]	37.5

Table S3. Results of the 2-step Individual Patient Data Meta-Analyses to Assess the Effect of 8q24.3 Copy Number Alterations on Prognosis.
Analysis were weighted by anatomical location, histological subtype or study, and presented as odds ratio's (OR) and 95% confidence intervals (CI).

Table S4

Cancer type	% died	% diploid	n	Model 1 (n=9,568) Deletion	Model 1 (n=9,568) Gain/amplification	Model 2 (n=7,593) Deletion	Model 2 (n=7,593) Gain/amplification
Bladder, urothelial carcinoma	44.8	39.1	87		0.84 [0.35-2.03]		
Brain, glioblastoma multiforme	79.6	81.7	540	0.98 [0.44-2.21]	0.91 [0.47-1.76]	0.98 [0.93-1.03]	1.01 [0.87-1.17]
Brain, lower grade glioma	19.6	78.5	480	1.68 [0.57-4.91]	0.78 [0.42-1.46]		
Breast, invasive carcinoma	8.2	58.9	343		1.21 [0.55-2.66]		
Breast, invasive ductal carcinoma	22.8	50.7	737	1.34 [0.51-3.52]	1.09 [0.77-1.55]		
Breast, invasive lobular carcinoma	22.8	74.0	127		0.95 [0.34-2.64]		
Breast, mixed ductal/lobular	18.5	64.6	65		2.14 [0.56-8.17]		
Cervix, squamous cell carcinoma and endocervical adenocarcinoma	19.9	52.5	276	0.78 [0.21-2.88]	1.14 [0.62-2.10]		
Colon, adenocarcinoma	19.4	36.0	314		1.38 [0.75-2.55]		1.59 [0.90-2.79]
Colorectal, mucinous adenocarcinoma	19.3	56.1	57		0.60 [0.14-2.62]		1.42 [0.23-8.95]
Embryonal carcinoma	5.3	10.5	19				
Esophagus, adenocarcinoma	42.3	33.8	71		2.83 [0.96-8.36]		
Esophagus, squamous cell carcinoma	35.2	18.2	88		0.59 [0.19-1.79]		
**Eye, uveal melanoma**	**26.7**	**24.0**	**75**		**8.50 [1.05-68.76]**		
Head and neck, squamous cell carcinoma	39.1	27.8	468		1.44 [0.94-2.21]		1.53 [0.94-2.48]
Hematological, acute myeloid leukemia	66.0	87.4	191		1.64 [0.62-4.35]		
Hematological/lymphoid, diffuse large B-cell lymphoma	17.0	66.0	47		0.76 [0.13-4.36]		
**Kidney, chromophobe renal cell carcinoma**	**8.6**	**60.3**	**58**		**1.52 [0.88-2.63]**		**1.38 [1.26-1.52]**
**Kidney, clear cell carcinoma**	**29.3**	**75.4**	**447**	**1.55 [0.17-13.84]**	**4.84 [1.96-11.92]**		
Kidney, renal papillary cell carcinoma	14.6	86.3	233				
Liver, hepatocellular carcinoma	32.6	31.9	313	1.49 [0.52-4.26]	1.01 [0.60-1.69]		
Lung, adenocarcinoma	32.3	37.8	400	1.85 [0.77-4.45]	1.51 [0.96-2.38]	2.02 [0.50-8.17]	1.51 [0.65-3.47]
**Lung, squamous cell carcinoma**	**35.0**	**28.0**	**411**	**1.11 [0.39-3.14]**	**1.62 [1.01-2.60]**	**1.79 [1.21-2.66]**	**1.92 [1.42-2.60]**
Mesothelioma	81.3	73.3	75		1.08 [0.26-4.48]		
**Ovaries, serous cystadenocarcinoma**	**47.5**	**16.1**	**503**	**1.81 [0.83-3.94]**	**1.18 [0.73-1.92]**	**1.83 [1.39-2.41]**	**1.44 [1.41-1.47]**
Pancreas, adenocarcinoma	55.8	60.4	154		1.83 [0.92-3.63]		
Pheochromocytoma	1.8	79.8	109		7.82 [0.46-134.08]		
**Prostate, adenocarcinoma**	**1.3**	**70.5**	**448**	**10.43 [1.00-109.3]**	**1.75 [0.29-10.63]**	**24.75 [0.80-765.6]**	**1.92 [1.61-2.30]**
Prostate, metastatic cancer	0.0	32.5	117				
Prostate, neuroendocrine cancer	0.0	23.3	82				
**Rectum, adenocarcinoma**	**17.6**	**35.2**	**142**		**3.04 [0.97-9.58]**		**2.43 [1.05-5.62]**
Skin, melanoma	31.6	42.1	335	1.22 [0.48-3.11]	0.95 [0.59-1.54]		
**Soft tissue sarcoma**	**33.5**	**55.1**	**185**	**1.47 [0.60-3.56]**	**2.05 [1.03-4.07]**		
Stomach, adenocarcinoma	34.9	35.2	261	0.53 [0.13-2.14]	0.66 [0.39-1.12]		
Stomach, adenocarcinoma diffuse type	39.1	43.5	69		0.91 [0.34-2.45]		1.05 [0.09-11.81]
**Stomach, tubular adenocarcinoma**	**38.8**	**23.9**	**67**		**0.23 [0.07-0.76]**		**0.21 [0.11-0.42]**
Testicles, non-seminomatous germ cell tumor	2.4	31.0	42				
Testicles, seminoma	0.0	14.1	64				
Thymus, thymoma	5.3	89.4	113		2.13 [0.22-20.30]		
Thyroid, follicular cancer	1.0	93.9	98				
Thyroid, papillary cancer	2.7	98.4	366				
Uterus, carcinosarcoma	60.0	17.8	45				
Uterus, endometrial carcinoma	17.7	29.4	17				
**Uterus, endometrioid carcinoma**	**10.9**	**72.8**	**357**		**2.68 [1.34-5.38]**		**3.63 [2.93-4.50]**
Uterus, serous carcinoma and papillary serous carcinoma	27.8	27.8	72	0.90 [0.17-4.64]	1.33 [0.39-4.51]	0.85 [0.45-1.58]	1.23 [0.32-4.67]
**Total**	**28.3**	**54.1**	**9,57**	**1.42 [1.16-1.73]**	**1.29 [1.17-1.41]**	**1.37 [0.88-2.15]**	**1.32 [0.78-2.25]**

Model 1 is unadjusted for any confounders, Model 2 is adjusted for age, sex, calendar period and clustering by study.

Table S4. Five-year Mortality by Copy Number Alteration using 8q24.3 Diploidy as a Reference. Table is categorised by histological subtypes of cancer, and expressed as odds ratios (OR) and 95% confidence intervals (CI).

15 cancer types with most cases of +1 and +2 8q24.3 copy number combined	Model 1		Model 2	
	Gain (+1)	Amplification (+2)	Gain (+1)	Amplification (+2)
Brain, lower grade glioma	0.67 [0.30-1.48]	1.01 [0.40-2.55]		
Breast, invasive carcinoma	1.50 [0.61-3.67]	0.88 [0.28-2.74]		
Breast, invasive ductal carcinoma	0.93 [0.59-1.46]	1.25 [0.83-1.89]		
Cervix, squamous cell carcinoma and endocervical adenocarcinoma	1.25 [0.68-2.32]			
**Colon, adenocarcinoma**	**1.36 [0.73-2.52]**	**1.76 [0.43-7.14]**	**1.58 [1.01-2.46]**	**1.57 [0.19-12.81]**
**Head and neck, squamous cell carcinoma**	**1.35 [0.88-2.09]**	**2.23 [1.10-4.51]**	**1.40 [0.75-2.62]**	**2.68 [2.17-3.30]**
Liver, hepatocellular carcinoma	0.90 [0.52-1.57]	1.31 [0.66-2.61]		
Lung, adenocarcinoma	1.57 [0.99-2.48]	0.99 [0.34-2.93]	1.57 [0.67-3.66]	0.92 [0.46-1.85]
**Lung, squamous cell carcinoma**	**1.79 [1.11-2.89]**	**0.62 [0.23-1.66]**	**2.07 [1.42-3.01]**	**0.88 [0.29-2.70]**
**Ovaries, serous cystadenocarcinoma**	**1.35 [0.81-2.27]**	**1.01 [0.60-1.72]**	**1.53 [1.15-2.05]**	**1.33 [0.91-1.93]**
**Prostate, adenocarcinoma**	**1.06 [0.11-10.35]**	**4.97 [0.50-49.85]**	**1.12 [0.76-1.64]**	**4.41 [3.41-5.71]**
**Rectum, adenocarcinoma**	**3.45 [1.09-10.89]**		**2.85 [1.20-6.75]**	
Skin, melanoma	0.91 [0.56-1.50]	1.22 [0.48-3.11]		
Stomach, adenocarcinoma	0.65 [0.38-1.12]	0.71 [0.20-2.53]		
**Uterus, endometrioid carcinoma**	**2.74 [1.35-5.57]**	**2.00 [0.23-17.44]**	**3.69 [3.46-3.93]**	**1.97 [0.11-34.35]**

Model 1 is unadjusted for any confounders, Model 2 is adjusted for age, sex, calendar period and clustering by study.

Table S5. Five-year Mortality of 15 cancer types with Most Cases of 8q24.3 Gain and Amplification using Diploidy as a Reference.

The risk of five-year mortality by copy number alterations, categorised by histological subtypes of cancer, and expressed as odds ratios (OR) and 95% confidence intervals (CI).

Supplemental appendix 1

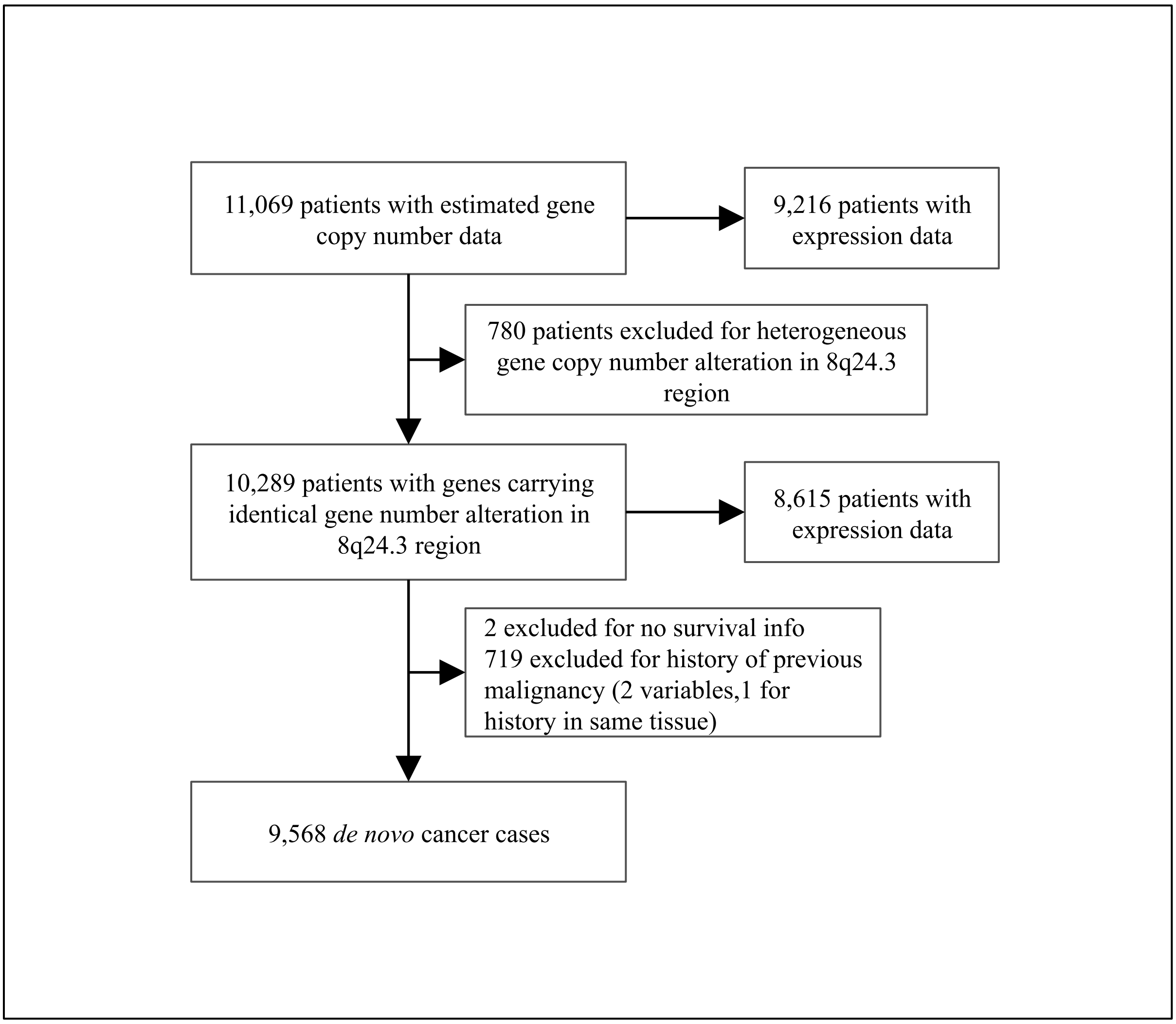

Appendix 1. Flow Chart Illustrating Selection of Individuals for Individual Patient Data Meta-Analysis.

Supplemental appendix 2

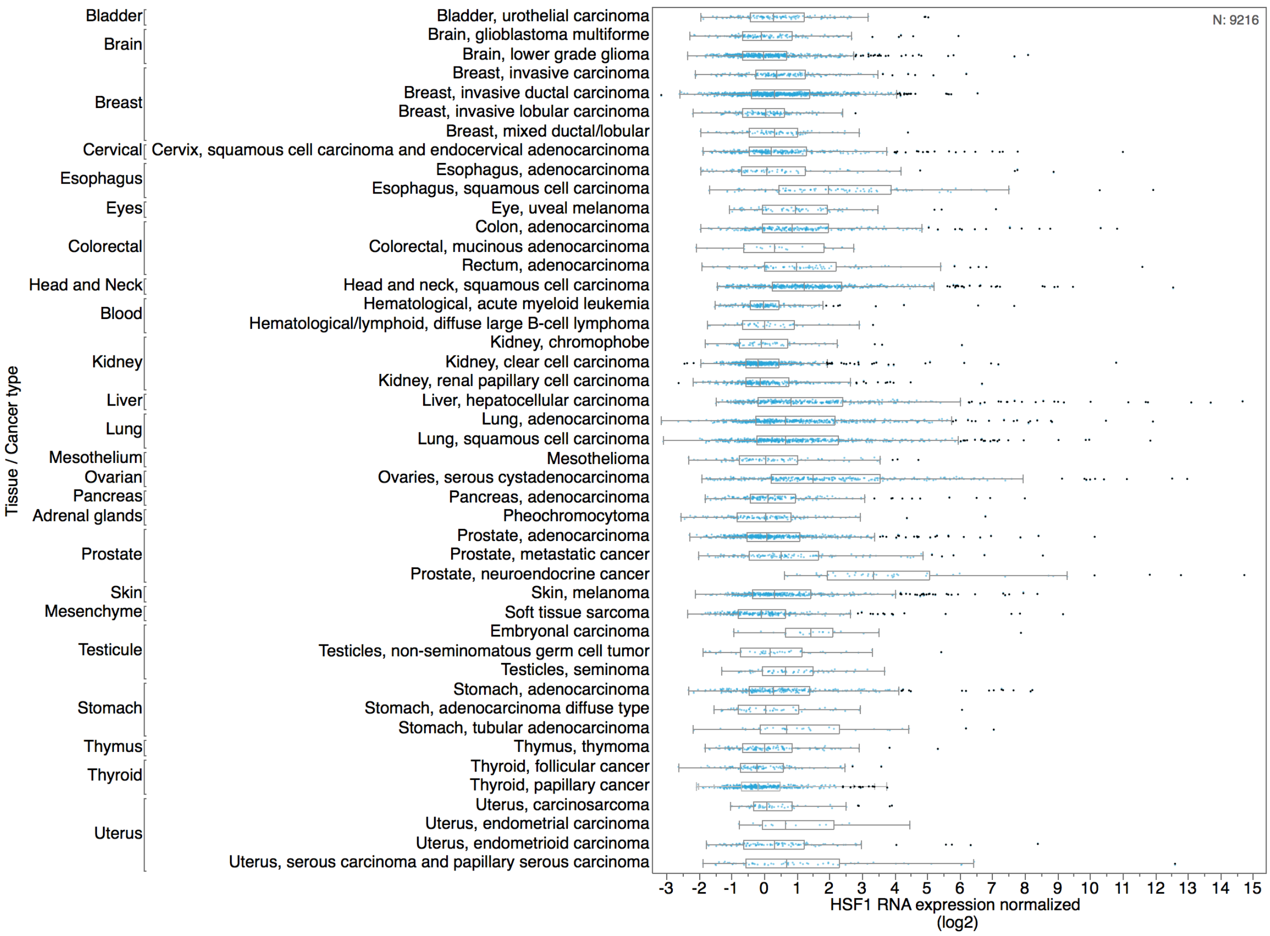

Appendix 2. Overview of HSF1 RNA Expression per Histological Subtype.
Normalised mRNA expression data of HSF1 (Z-scores 2) are represented in blue dots. Box plots show additionally the median values at centred lines; box limits indicate the 25th and 75th percentiles; whiskers extend 1.5 times the interquartile range from the 25th and 75th percentiles; outliers are represented in black dots.

Supplemental appendix 3

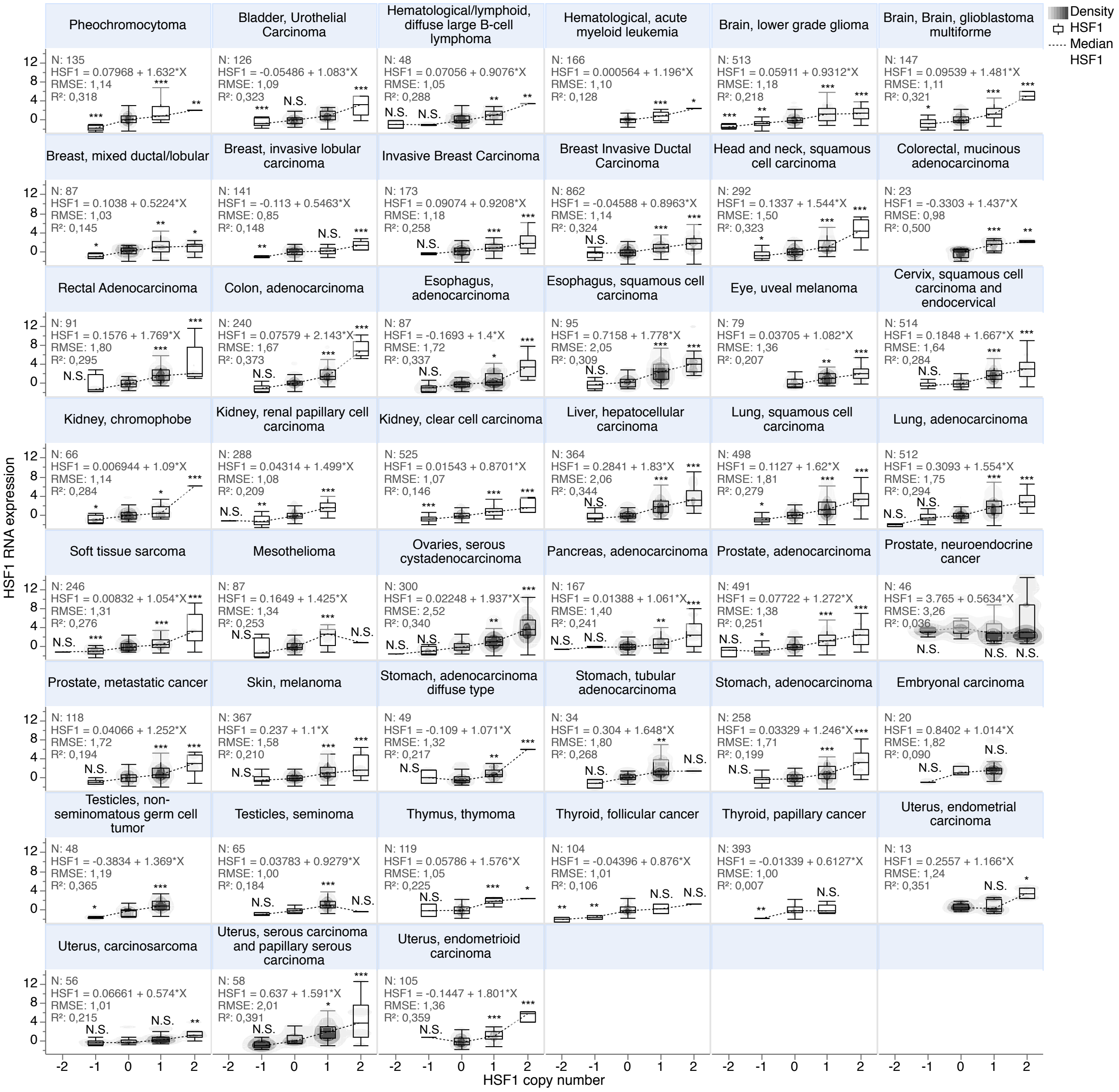

Appendix 3. Expression of HSF1 in Function of HSF1 Copy Number Alteration per Histological Subtype.

Box plots show the median values at the centred lines; box limits indicate the 25th and 75th percentiles; whiskers extend 1.5 times the interquartile range from the 25th and 75th percentiles. Dashed lines rely medians of each copy number category. Grey contour shows regions of density data. Caption boxes indicate N = number of patient, linear equations indicate in function of HSF1 expression (linear lines not show in the graphs), RMSE = root-mean-square error, R2 = determination coefficient. P-values were calculated using a two-tailed unpaired t-test compared to the diploid (i.e. 0). N.S., not significant (i.e. p>0.05), *p< 0.05, **p<0.01, ***p<0.001

Supplemental appendix 4 (1 of 2)

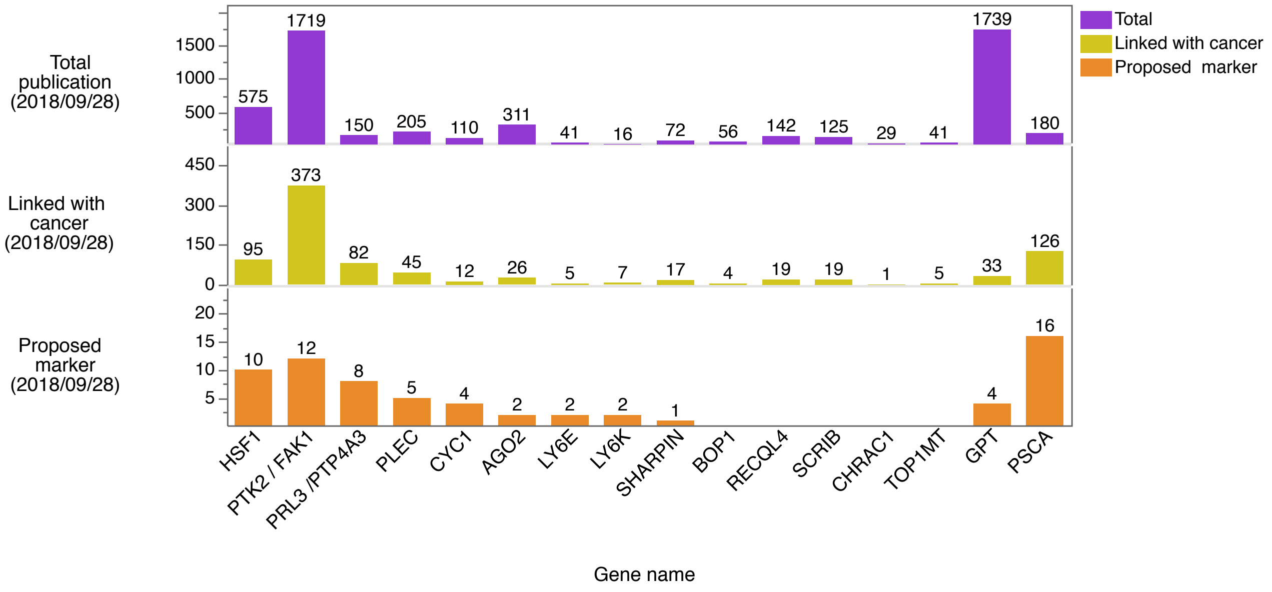

Appendix 4. Number of publication of selected popular cancer-related genes per field located in 8q24.3 cytoband.

Publication finding has been performed using GeneCard until the September 28th 2018. Top bar graph indicates the total number of publications per gene. Middle graph shows the number of publications associated in cancer (cancer model or cancer research) for each gene. Bottom graph displays the amount of publications where genes are suggested to be use as a biomarker.

Supplemental appendix 4 (2 of 2)

Gene name	Gene description	Location	Gene type	Entrez gene ID	Publications (2018/09/28) Total	Linked with cancer	Proposed marker	GeneCards Summary
HSF1	Heat Shock Transcription Factor 1	8q24.3	Protein Coding	3297	575	95	10	HSF1 (Heat Shock Transcription Factor 1) is a Protein Coding gene. Diseases associated with HSF1 include Heat-Shock Rna 1 and Synucleinopathy. Among its related pathways are Legionellosis and Sertoli-Sertoli Cell Junction Dynamics. Gene Ontology (GO) annotations related to this gene include DNA binding transcription factor activity and chromatin binding. An important paralog of this gene is HSF2.
PTK2 / FAK1	Protein Tyrosine Kinase 2	8q24.3	Protein coding	5747	1719	373	12	PTK2 (Protein Tyrosine Kinase 2) is a Protein Coding gene. Diseases associated with PTK2 include Glioblastoma Multiforme and Colorectal Cancer. Among its related pathways are ERK Signaling and Focal Adhesion. Gene Ontology (GO) annotations related to this gene include transferase activity, transferring phosphorus-containing groups and protein tyrosine kinase activity. An important paralog of this gene is PTK2B.
PRL3 /PTP4A3	Protein Tyrosine Phosphatase Type IVA, Member 3	8q24.3	Protein coding	11156	150	82	8	PTP4A3 (Protein Tyrosine Phosphatase Type IVA, Member 3) is a Protein Coding gene. Among its related pathways are Signaling events mediated by PRL and PAK Pathway. Gene Ontology (GO) annotations related to this gene include phosphatase activity and protein tyrosine/serine/threonine phosphatase activity. An important paralog of this gene is PTP4A1.
PLEC	Plectin	8q24.3	Protein Coding	5339	205	45	5	PLEC (Plectin) is a Protein Coding gene. Diseases associated with PLEC include Epidermolysis Bullosa Simplex, Ogna Type and Muscular Dystrophy, Limb-Girdle, Type 2Q. Among its related pathways are Collagen chain trimerization and Cytoskeletal Signaling. Gene Ontology (GO) annotations related to this gene include structural constituent of muscle. An important paralog of this gene is MACF1.
CYC1	Cytochrome C1	8q24.3	Protein Coding	1537	110	12	4	CYC1 (Cytochrome C1) is a Protein Coding gene. Diseases associated with CYC1 include Mitochondrial Complex Iii Deficiency, Nuclear Type 6 and Isolated Complex Iii Deficiency. Among its related pathways are Metabolism and Respiratory electron transport, ATP synthesis by chemiosmotic coupling, and heat production by uncoupling proteins.. Gene Ontology (GO) annotations related to this gene include iron ion binding and electron transfer activity.
AGO2	Argonaute 2, RISC Catalytic Component	8q24.3	Protein coding	27161	311	26	2	AGO2 (Argonaute 2, RISC Catalytic Component) is a Protein Coding gene. Diseases associated with AGO2 include Colorectal Cancer and Chromosome 18P Deletion Syndrome. Among its related pathways are Gene Expression and Mitotic Prophase. Gene Ontology (GO) annotations related to this gene include nucleic acid binding and RNA binding. An important paralog of this gene is AGO1.
LY6E	Lymphocyte Antigen 6 Family Member E	8q24.3	Protein Coding	4061	41	5	2	LY6E (Lymphocyte Antigen 6 Family Member E) is a Protein Coding gene. Diseases associated with LY6E include Acute Promyelocytic Leukemia. Among its related pathways are Metabolism of proteins and Post-translational modification- synthesis of GPI-anchored proteins.
LY6K	Lymphocyte Antigen 6 Family Member K	8q24.3	Protein Coding	54742	16	7	2	LY6K (Lymphocyte Antigen 6 Family Member K) is a Protein Coding gene. Diseases associated with LY6K include Cyclothymic Disorder. Among its related pathways are Metabolism of proteins and Post-translational modification- synthesis of GPI-anchored proteins.
SHARPIN	SHANK Associated RH Domain Interactor	8q24.3	Protein Coding	81858	72	17	1	SHARPIN (SHANK Associated RH Domain Interactor) is a Protein Coding gene. Diseases associated with SHARPIN include Glycogen Storage Disease Iv. Among its related pathways are TNF signaling (REACTOME) and Protein-protein interactions at synapses. Gene Ontology (GO) annotations related to this gene include identical protein binding and polyubiquitin modification-dependent protein binding. An important paralog of this gene is RBCK1.
BOP1	Block Of Proliferation 1	8q24.3	Protein Coding	23246	56	4	0	BOP1 (Block Of Proliferation 1) is a Protein Coding gene. Among its related pathways are rRNA processing in the nucleus and cytosol and Gene Expression. Gene Ontology (GO) annotations related to this gene include ribonucleoprotein complex binding.
RECQL4	RecQ Like Helicase 4	8q24.3	Protein Coding	9401	142	19	0	RECQL4 (RecQ Like Helicase 4) is a Protein Coding gene. Diseases associated with RECQL4 include Baller-Gerold Syndrome and Rapadilino Syndrome. Among its related pathways are DNA Damage. Gene Ontology (GO) annotations related to this gene include nucleic acid binding and annealing helicase activity. An important paralog of this gene is BLM.
SCRIB	Scribbled Planar Cell Polarity Protein	8q24.3	Protein Coding	23513	125	19	0	SCRIB (Scribbled Planar Cell Polarity Protein) is a Protein Coding gene. Diseases associated with SCRIB include Neural Tube Defects and Tick-Borne Encephalitis. Among its related pathways are Cytoskeletal Signaling and Signaling by Wnt. An important paralog of this gene is LRRC7.
CHRAC1	Chromatin Accessibility Complex 1	8q24.3	Protein coding	54108	29	1	0	CHRAC1 (Chromatin Accessibility Complex 1) is a Protein Coding gene. Gene Ontology (GO) annotations related to this gene include sequence-specific DNA binding and DNA-directed DNA polymerase activity.
TOP1MT	DNA Topoisomerase I Mitochondrial	8q24.3	Protein Coding	116447	41	5	0	TOP1MT (DNA Topoisomerase I Mitochondrial) is a Protein Coding gene. Gene Ontology (GO) annotations related to this gene include DNA topoisomerase type I activity and DNA topoisomerase type II (ATP-hydrolyzing) activity. An important paralog of this gene is TOP1.
GPT	Glutamic--Pyruvic Transaminase	8q24.3	Protein Coding	2875	1739	33	4	GPT (Glutamic--Pyruvic Transaminase) is a Protein Coding gene. Diseases associated with GPT include Hepatitis and Choledocholithiasis. Among its related pathways are Cori Cycle and Alanine and aspartate metabolism. Gene Ontology (GO) annotations related to this gene include pyridoxal phosphate binding and L-alanine:2-oxoglutarate aminotransferase activity. An important paralog of this gene is GPT2.
PSCA	Prostate Stem Cell Antigen	8q24.3	Protein Coding	8000	180	126	16	PSCA (Prostate Stem Cell Antigen) is a Protein Coding gene. Diseases associated with PSCA include Colloid Carcinoma Of The Pancreas and Gallbladder Adenocarcinoma. Among its related pathways are Metabolism of proteins and Post-translational modification- synthesis of GPI-anchored proteins.
			**Total publication**		**5511**	**869**	**66**	

Appendix 4. Description of selected cancer-related genes per field located in 8q24.3 cytoband.
Descriptive table for popular cancer-related genes per field located in 8q24.3 cytoband. Description summary were obtained from GeneCards

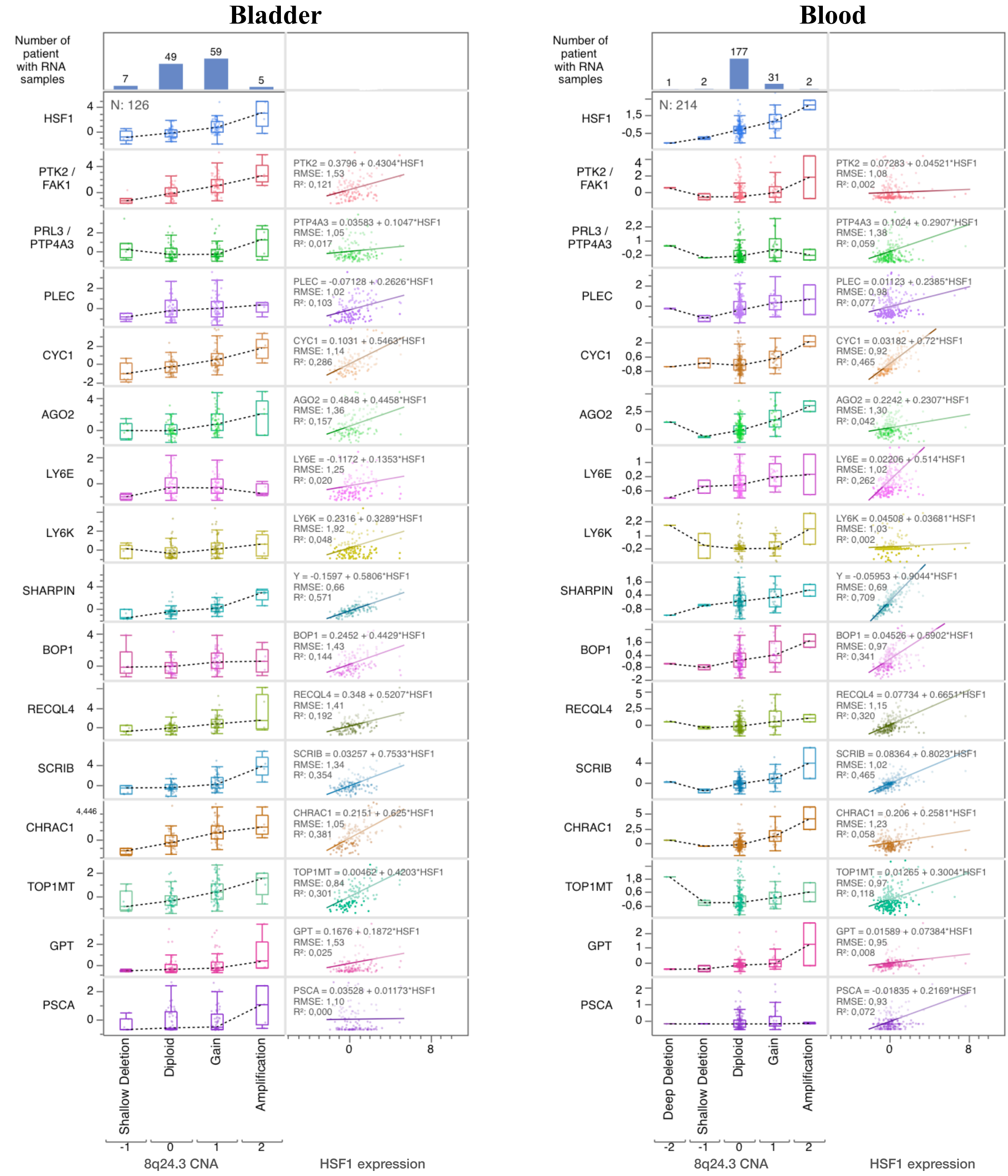

Appendix 5. Influence of 8q24.3 Copy Number on other Cancer-Related Genes Expression Located in 8q24.3 Loci and Comparison with HSF1 Related Expression per Tissue.

Left panel displays the HSF1 RNA expression in function of 8q24.3 copy number. Box plots for each graph of each gene described in **appendix 4** show the median values at the centred lines; box limits indicate the 25th and 75th percentiles; whiskers extend 1.5 times the interquartile range from the 25th and 75th percentiles. Dashed lines rely medians of each copy number category. N = number of patient in each 8q24.3 copy number alteration category are shown in bar graph in top left panel. Right panel displays the RNA expression of the indicated gene in function of HSF1 RNA expression. Each dot represents a patient data. Linear trends are indicated and linear equations are displayed in the caption box for each gene. RMSE = root-mean-square error, R^2 = determination coefficient.

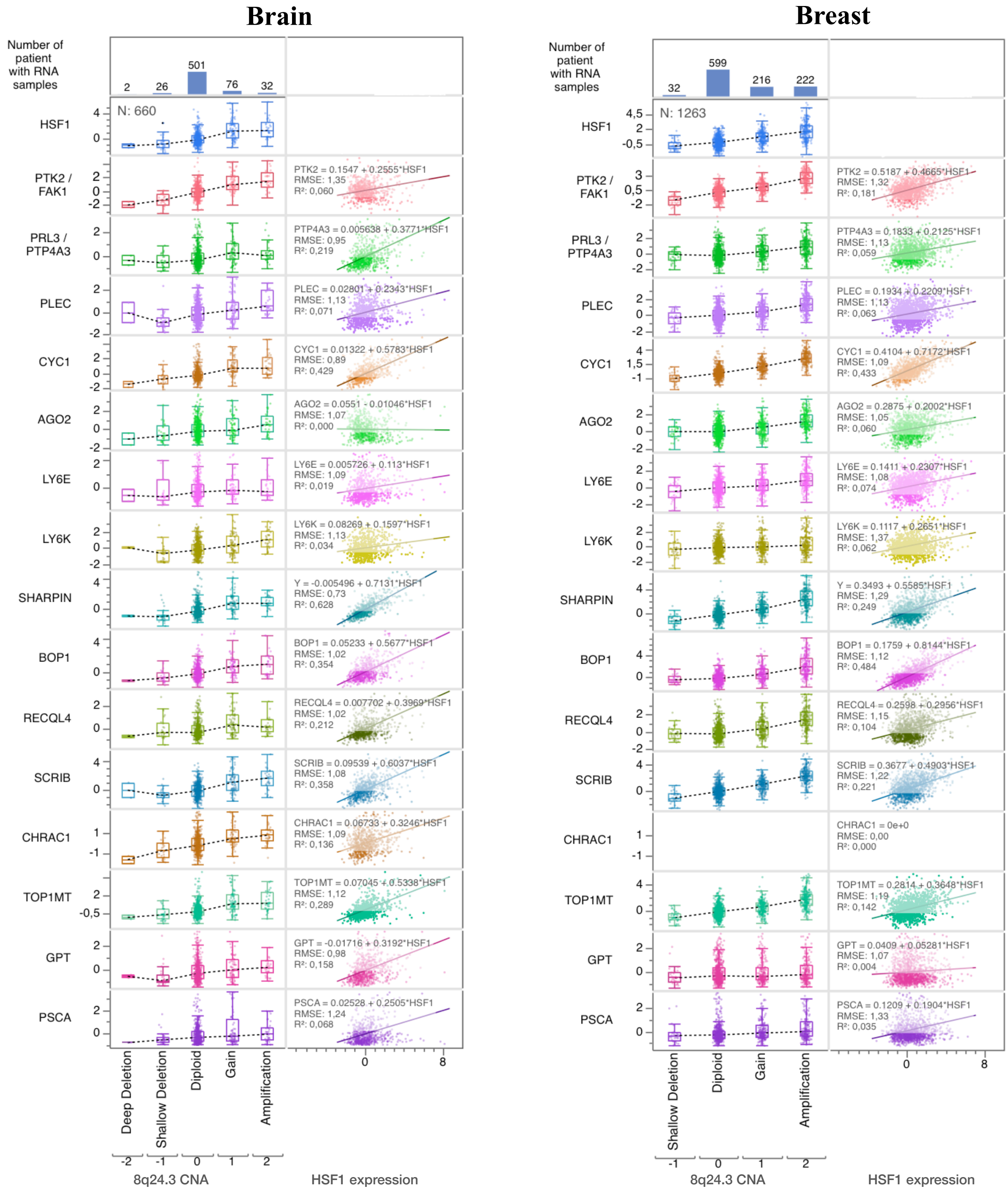

Appendix 5. Influence of 8q24.3 Copy Number on other Cancer-Related Genes Expression Located in 8q24.3 Loci and Comparison with HSF1 Related Expression per Tissue.

Left panel displays the HSF1 RNA expression in function of 8q24.3 copy number. Box plots for each graph of each gene described in **appendix 4** show the median values at the centred lines; box limits indicate the 25th and 75th percentiles; whiskers extend 1.5 times the interquartile range from the 25th and 75th percentiles. Dashed lines rely medians of each copy number category. N = number of patient in each 8q24.3 copy number alteration category are shown in bar graph in top left panel. Right panel displays the RNA expression of the indicated gene in function of HSF1 RNA expression. Each dot represents a patient data. Linear trends are indicated and linear equations are displayed in the caption box for each gene. RMSE = root-mean-square error, R^2 = determination coefficient.

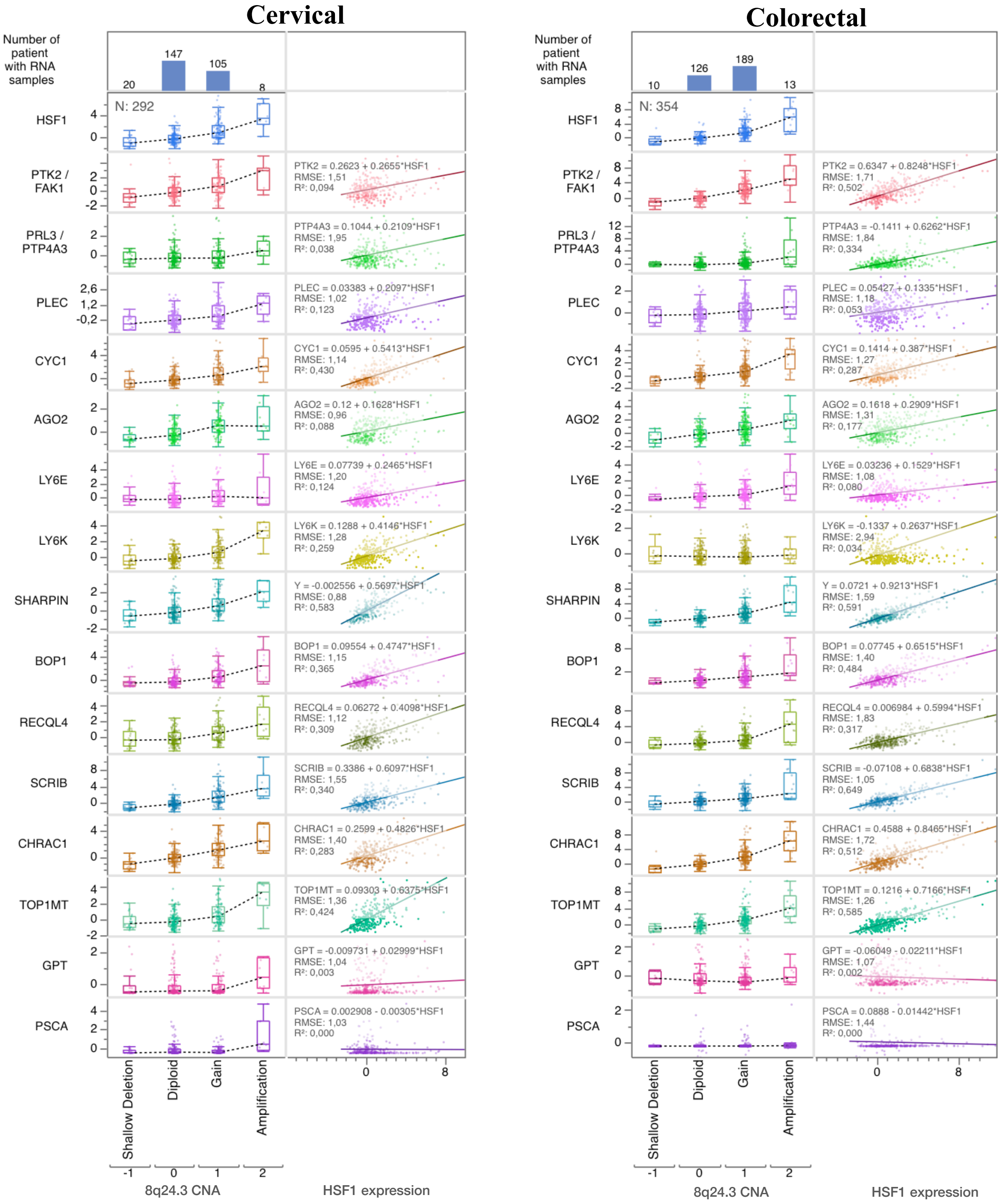

Appendix 5. Influence of 8q24.3 Copy Number on other Cancer-Related Genes Expression Located in 8q24.3 Loci and Comparison with HSF1 Related Expression per Tissue.

Left panel displays the HSF1 RNA expression in function of 8q24.3 copy number. Box plots for each graph of each gene described in **appendix 4** show the median values at the centred lines; box limits indicate the 25th and 75th percentiles; whiskers extend 1.5 times the interquartile range from the 25th and 75th percentiles. Dashed lines rely medians of each copy number category. N = number of patient in each 8q24.3 copy number alteration category are shown in bar graph in top left panel. Right panel displays the RNA expression of the indicated gene in function of HSF1 RNA expression. Each dot represents a patient data. Linear trends are indicated and linear equations are displayed in the caption box for each gene. RMSE = root-mean-square error, R^2 = determination coefficient.

Oesophagus **Eyes**

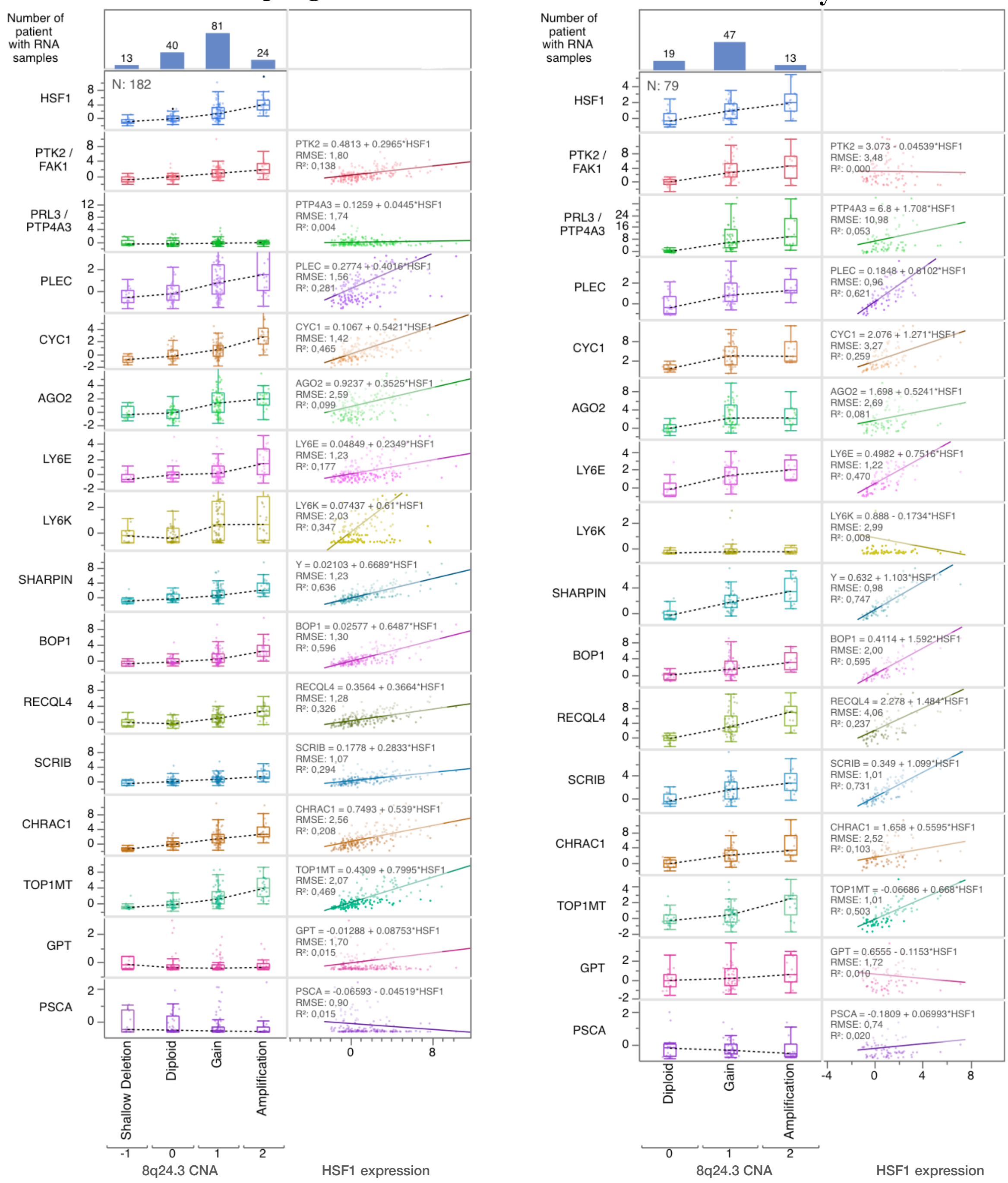

Appendix 5. Influence of 8q24.3 Copy Number on other Cancer-Related Genes Expression Located in 8q24.3 Loci and Comparison with HSF1 Related Expression per Tissue.
Left panel displays the HSF1 RNA expression in function of 8q24.3 copy number. Box plots for each graph of each gene described in **appendix 4** show the median values at the centred lines; box limits indicate the 25th and 75th percentiles; whiskers extend 1.5 times the interquartile range from the 25th and 75th percentiles. Dashed lines rely medians of each copy number category. N = number of patient in each 8q24.3 copy number alteration category are shown in bar graph in top left panel. Right panel displays the RNA expression of the indicated gene in function of HSF1 RNA expression. Each dot represents a patient data. Linear trends are indicated and linear equations are displayed in the caption box for each gene. RMSE = root-mean-square error, R^2 = determination coefficient.

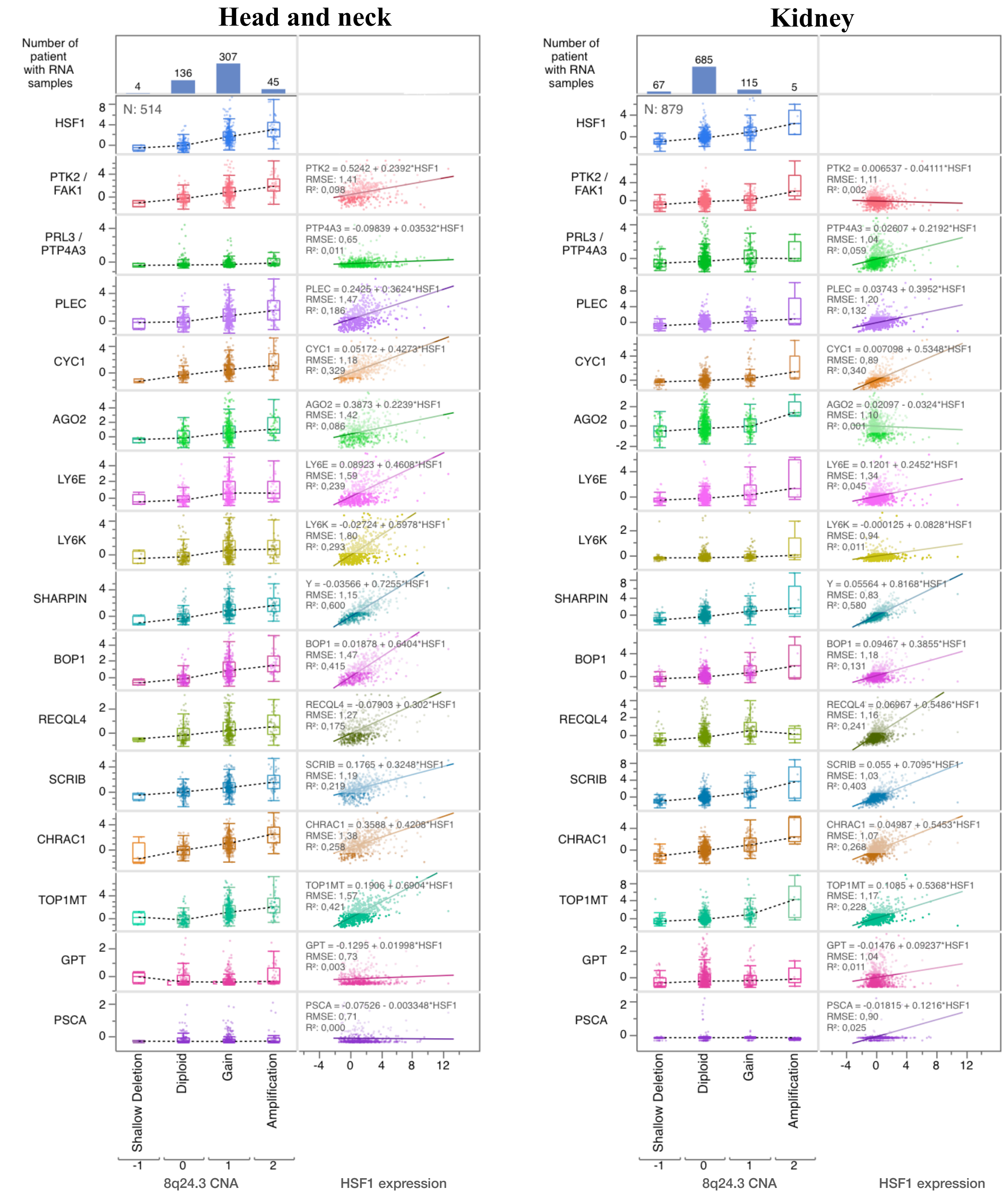

Appendix 5. Influence of 8q24.3 Copy Number on other Cancer-Related Genes Expression Located in 8q24.3 Loci and Comparison with HSF1 Related Expression per Tissue.
Left panel displays the HSF1 RNA expression in function of 8q24.3 copy number. Box plots for each graph of each gene described in **appendix 4** show the median values at the centred lines; box limits indicate the 25th and 75th percentiles; whiskers extend 1.5 times the interquartile range from the 25th and 75th percentiles. Dashed lines rely medians of each copy number category. N = number of patient in each 8q24.3 copy number alteration category are shown in bar graph in top left panel. Right panel displays the RNA expression of the indicated gene in function of HSF1 RNA expression. Each dot represents a patient data. Linear trends are indicated and linear equations are displayed in the caption box for each gene. RMSE = root-mean-square error, R^2 = determination coefficient.

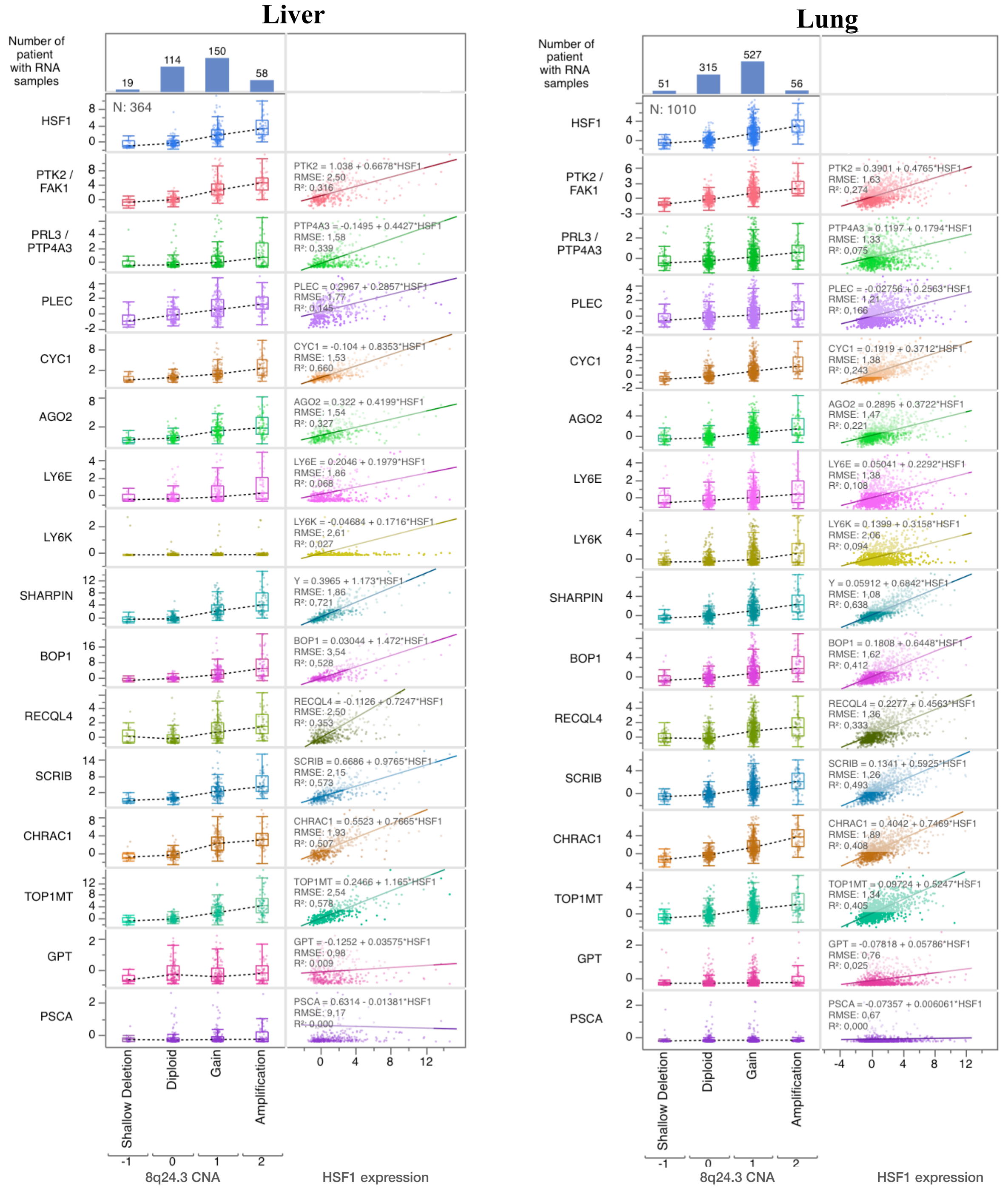

Appendix 5. Influence of 8q24.3 Copy Number on other Cancer-Related Genes Expression Located in 8q24.3 Loci and Comparison with HSF1 Related Expression per Tissue.

<u>Left panel</u> displays the HSF1 RNA expression in function of 8q24.3 copy number. Box plots for each graph of each gene described in **appendix 4** show the median values at the centred lines; box limits indicate the 25th and 75th percentiles; whiskers extend 1.5 times the interquartile range from the 25th and 75th percentiles. Dashed lines rely medians of each copy number category. N = number of patient in each 8q24.3 copy number alteration category are shown in bar graph in top left panel. <u>Right panel</u> displays the RNA expression of the indicated gene in function of HSF1 RNA expression. Each dot represents a patient data. Linear trends are indicated and linear equations are displayed in the caption box for each gene. RMSE = root-mean-square error, R^2 = determination coefficient.

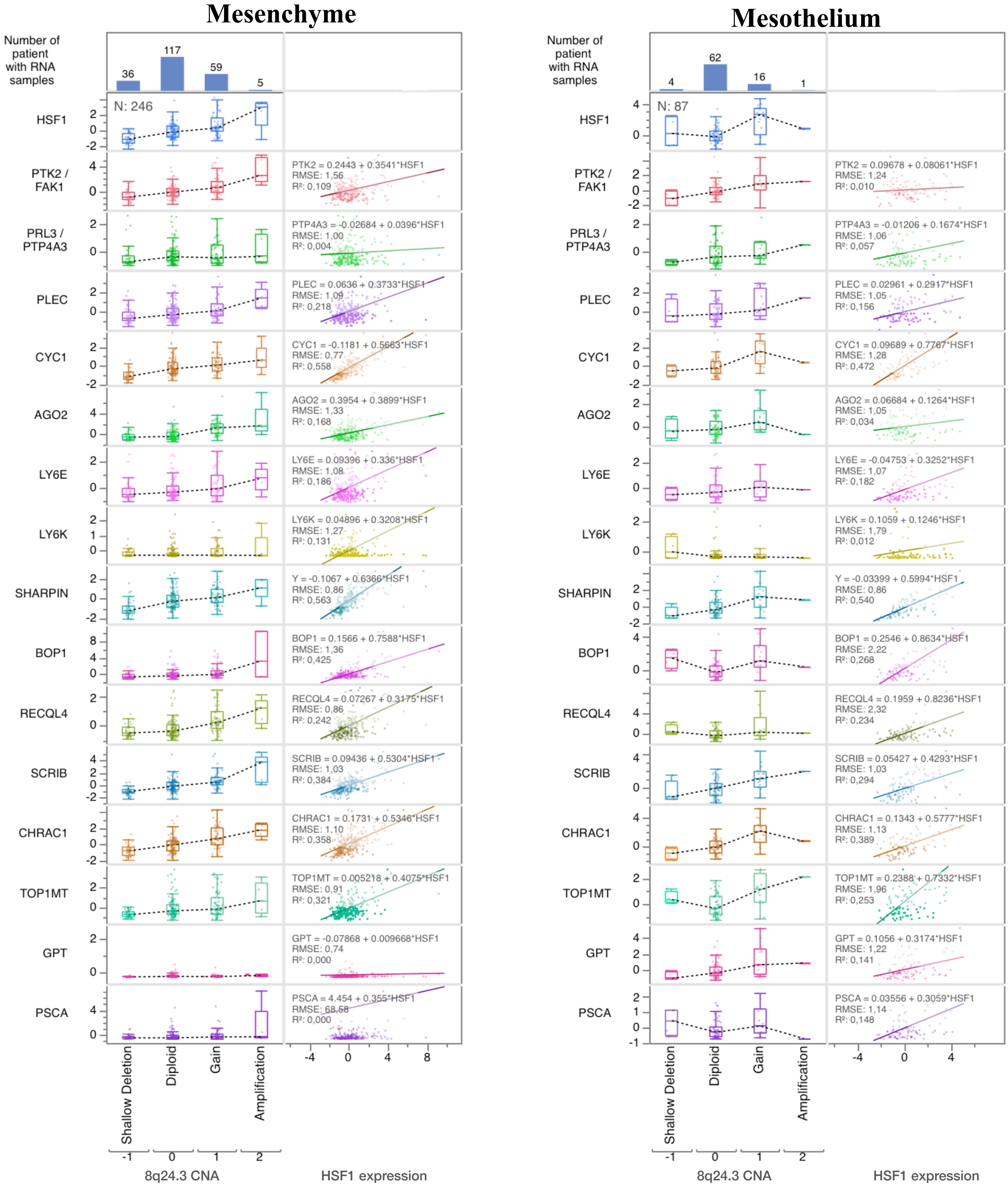

Appendix 5. Influence of 8q24.3 Copy Number on other Cancer-Related Genes Expression Located in 8q24.3 Loci and Comparison with HSF1 Related Expression per Tissue.

Left panel displays the HSF1 RNA expression in function of 8q24.3 copy number. Box plots for each graph of each gene described in **appendix 4** show the median values at the centred lines; box limits indicate the 25th and 75th percentiles; whiskers extend 1.5 times the interquartile range from the 25th and 75th percentiles. Dashed lines rely medians of each copy number category. N = number of patient in each 8q24.3 copy number alteration category are shown in bar graph in top left panel. Right panel displays the RNA expression of the indicated gene in function of HSF1 RNA expression. Each dot represents a patient data. Linear trends are indicated and linear equations are displayed in the caption box for each gene. RMSE = root-mean-square error, R^2 = determination coefficient.

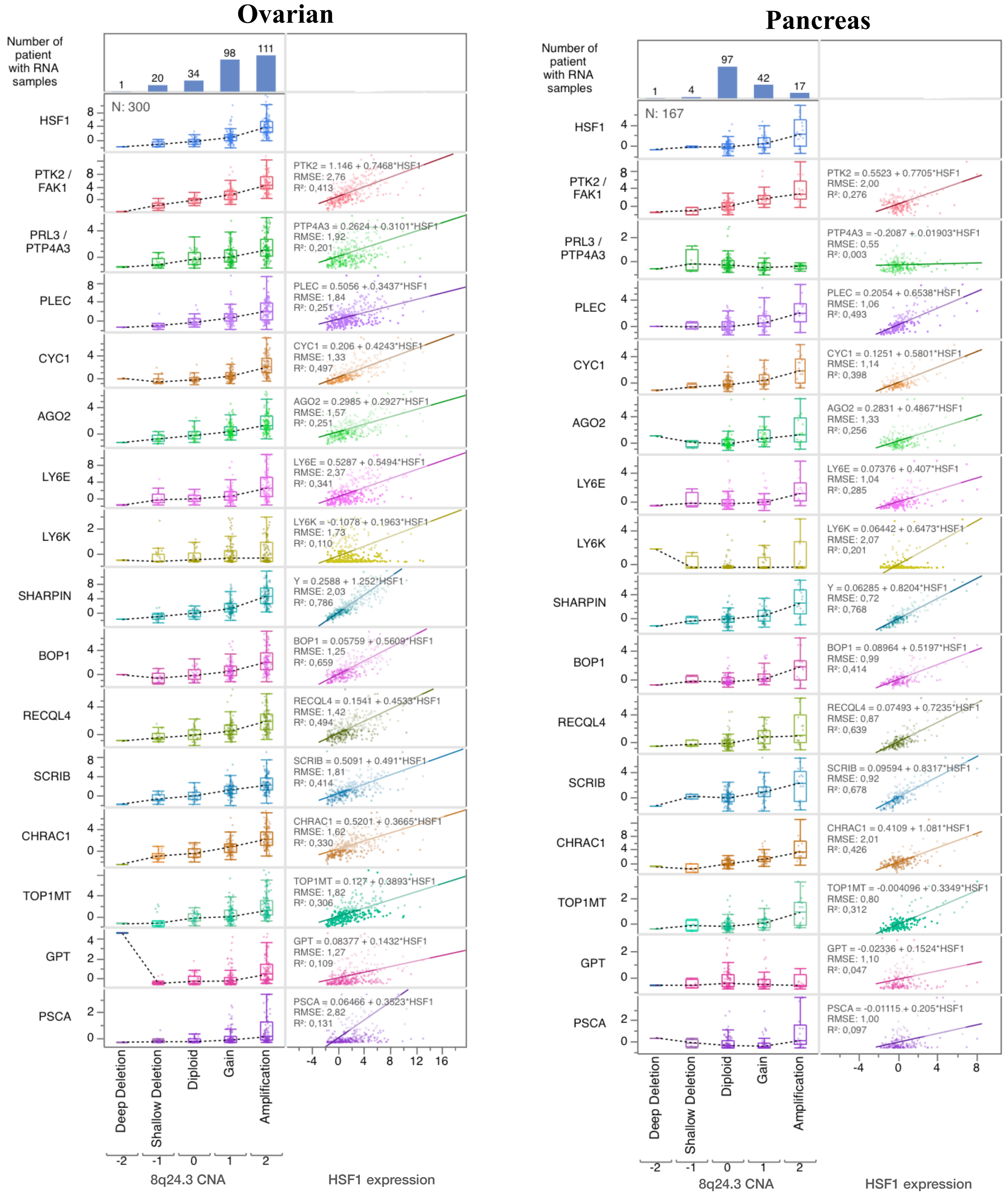

Appendix 5. Influence of 8q24.3 Copy Number on other Cancer-Related Genes Expression Located in 8q24.3 Loci and Comparison with HSF1 Related Expression per Tissue.

<u>Left panel</u> displays the HSF1 RNA expression in function of 8q24.3 copy number. Box plots for each graph of each gene described in **appendix 4** show the median values at the centred lines; box limits indicate the 25th and 75th percentiles; whiskers extend 1.5 times the interquartile range from the 25th and 75th percentiles. Dashed lines rely medians of each copy number category. N = number of patient in each 8q24.3 copy number alteration category are shown in bar graph in top left panel. <u>Right panel</u> displays the RNA expression of the indicated gene in function of HSF1 RNA expression. Each dot represents a patient data. Linear trends are indicated and linear equations are displayed in the caption box for each gene. RMSE = root-mean-square error, R^2 = determination coefficient.

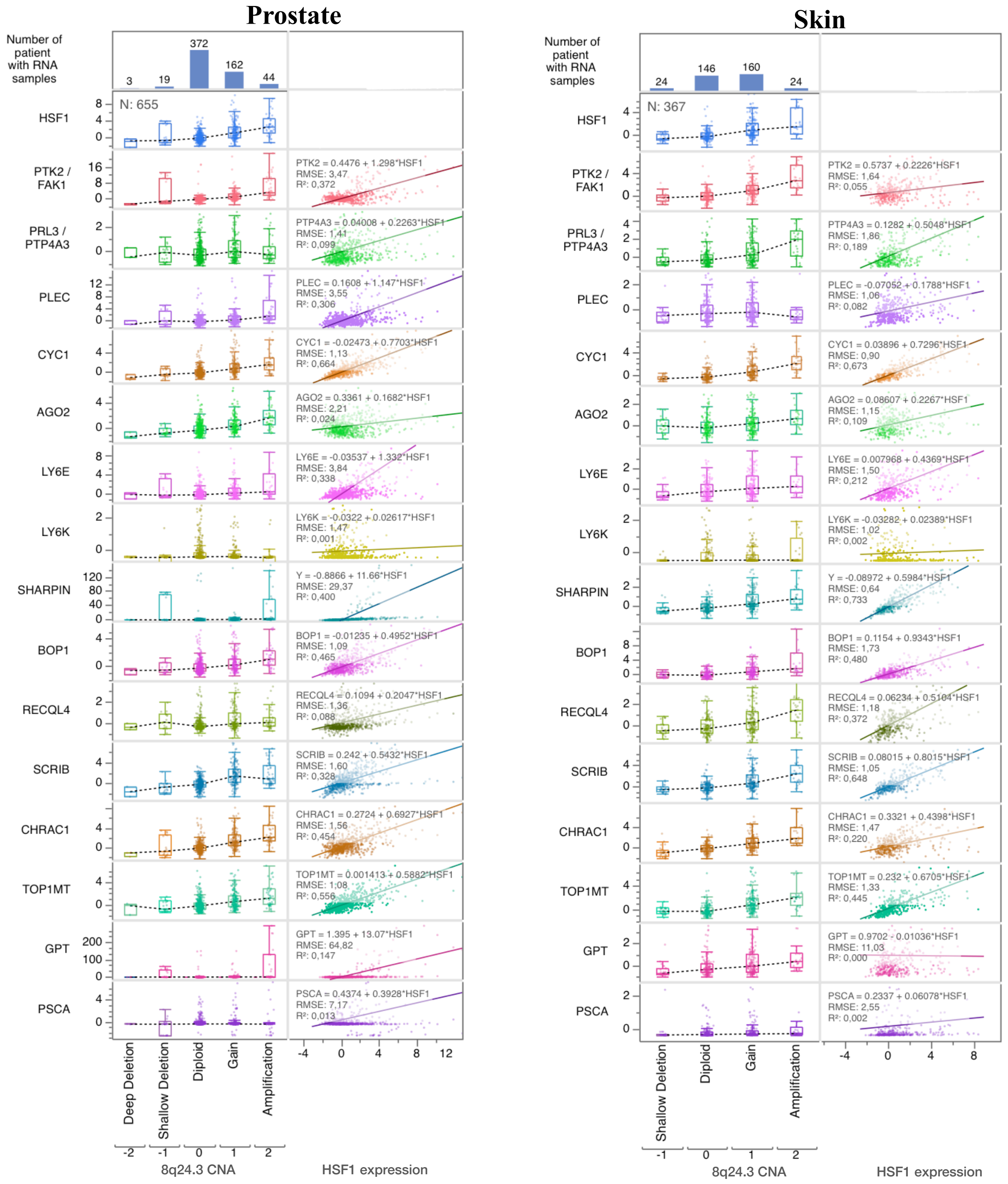

Appendix 5. Influence of 8q24.3 Copy Number on other Cancer-Related Genes Expression Located in 8q24.3 Loci and Comparison with HSF1 Related Expression per Tissue.

Left panel displays the HSF1 RNA expression in function of 8q24.3 copy number. Box plots for each graph of each gene described in **appendix 4** show the median values at the centred lines; box limits indicate the 25th and 75th percentiles; whiskers extend 1.5 times the interquartile range from the 25th and 75th percentiles. Dashed lines rely medians of each copy number category. N = number of patient in each 8q24.3 copy number alteration category are shown in bar graph in top left panel. Right panel displays the RNA expression of the indicated gene in function of HSF1 RNA expression. Each dot represents a patient data. Linear trends are indicated and linear equations are displayed in the caption box for each gene. RMSE = root-mean-square error, R^2 = determination coefficient.

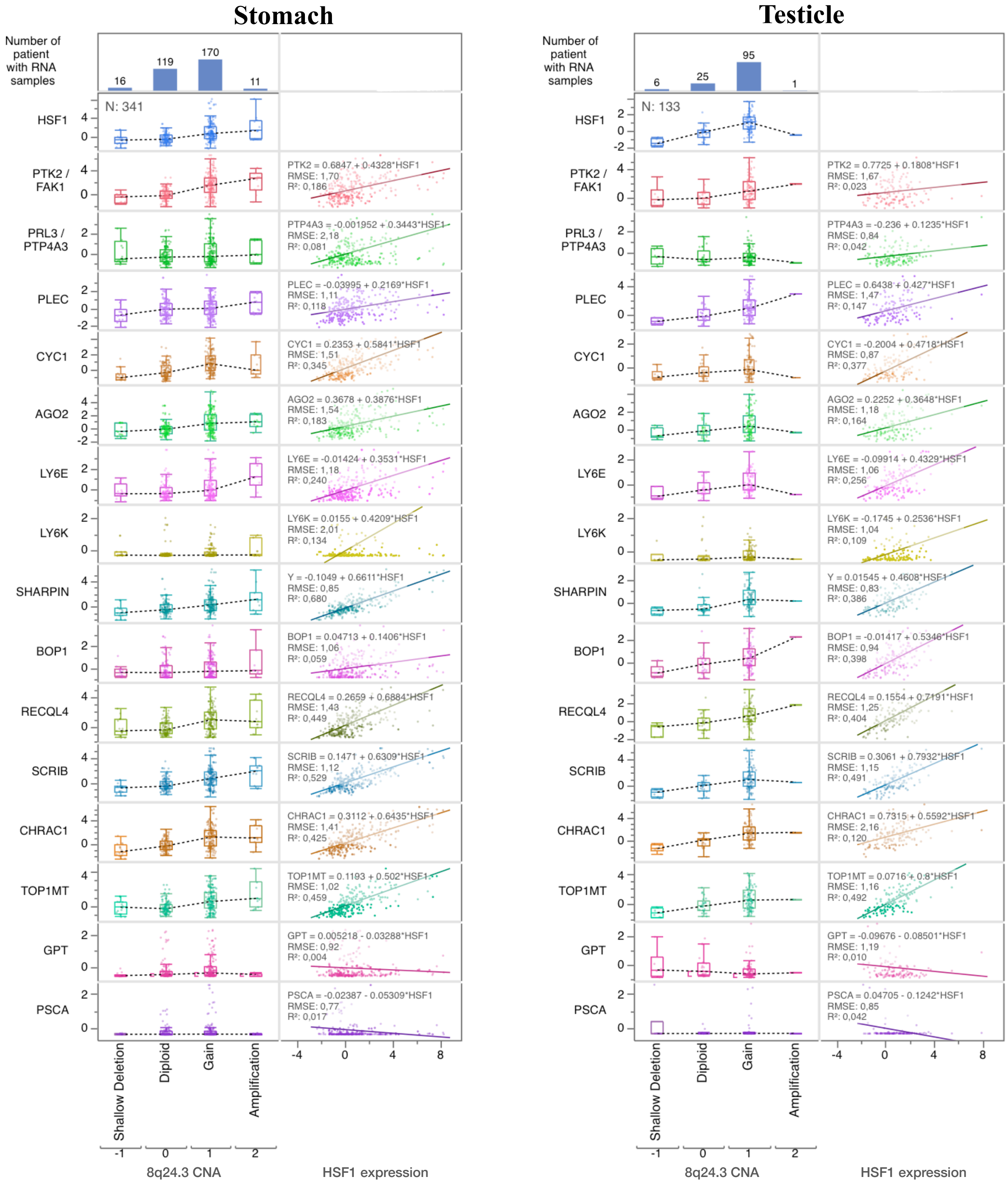

Appendix 5. Influence of 8q24.3 Copy Number on other Cancer-Related Genes Expression Located in 8q24.3 Loci and Comparison with HSF1 Related Expression per Tissue.
Left panel displays the HSF1 RNA expression in function of 8q24.3 copy number. Box plots for each graph of each gene described in **appendix 4** show the median values at the centred lines; box limits indicate the 25th and 75th percentiles; whiskers extend 1.5 times the interquartile range from the 25th and 75th percentiles. Dashed lines rely medians of each copy number category. N = number of patient in each 8q24.3 copy number alteration category are shown in bar graph in top left panel. Right panel displays the RNA expression of the indicated gene in function of HSF1 RNA expression. Each dot represents a patient data. Linear trends are indicated and linear equations are displayed in the caption box for each gene. RMSE = root-mean-square error, R^2 = determination coefficient.

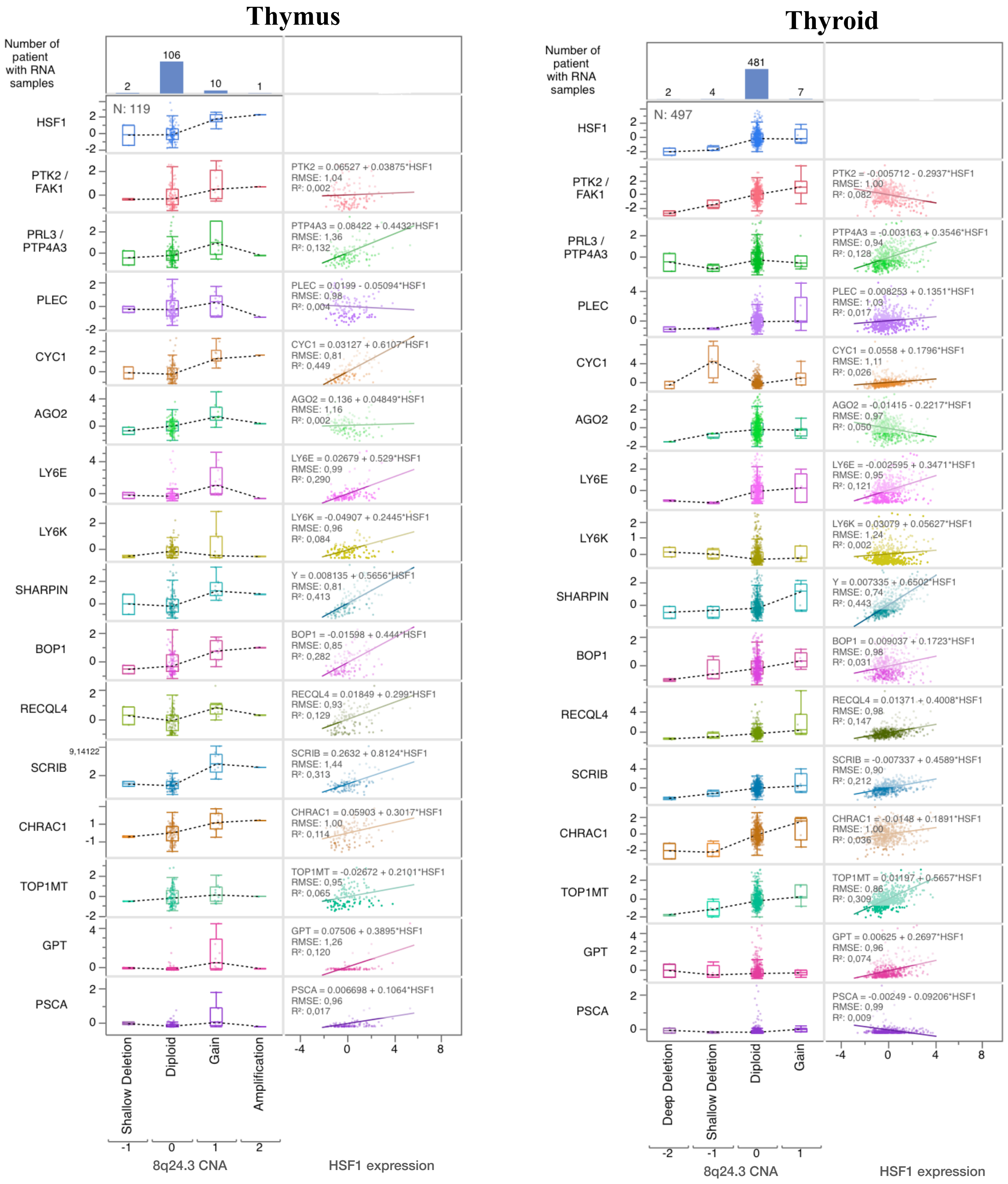

Appendix 5. Influence of 8q24.3 Copy Number on other Cancer-Related Genes Expression Located in 8q24.3 Loci and Comparison with HSF1 Related Expression per Tissue.

Left panel displays the HSF1 RNA expression in function of 8q24.3 copy number. Box plots for each graph of each gene described in **appendix 4** show the median values at the centred lines; box limits indicate the 25th and 75th percentiles; whiskers extend 1.5 times the interquartile range from the 25th and 75th percentiles. Dashed lines rely medians of each copy number category. N = number of patient in each 8q24.3 copy number alteration category are shown in bar graph in top left panel. Right panel displays the RNA expression of the indicated gene in function of HSF1 RNA expression. Each dot represents a patient data. Linear trends are indicated and linear equations are displayed in the caption box for each gene. RMSE = root-mean-square error, R^2 = determination coefficient.

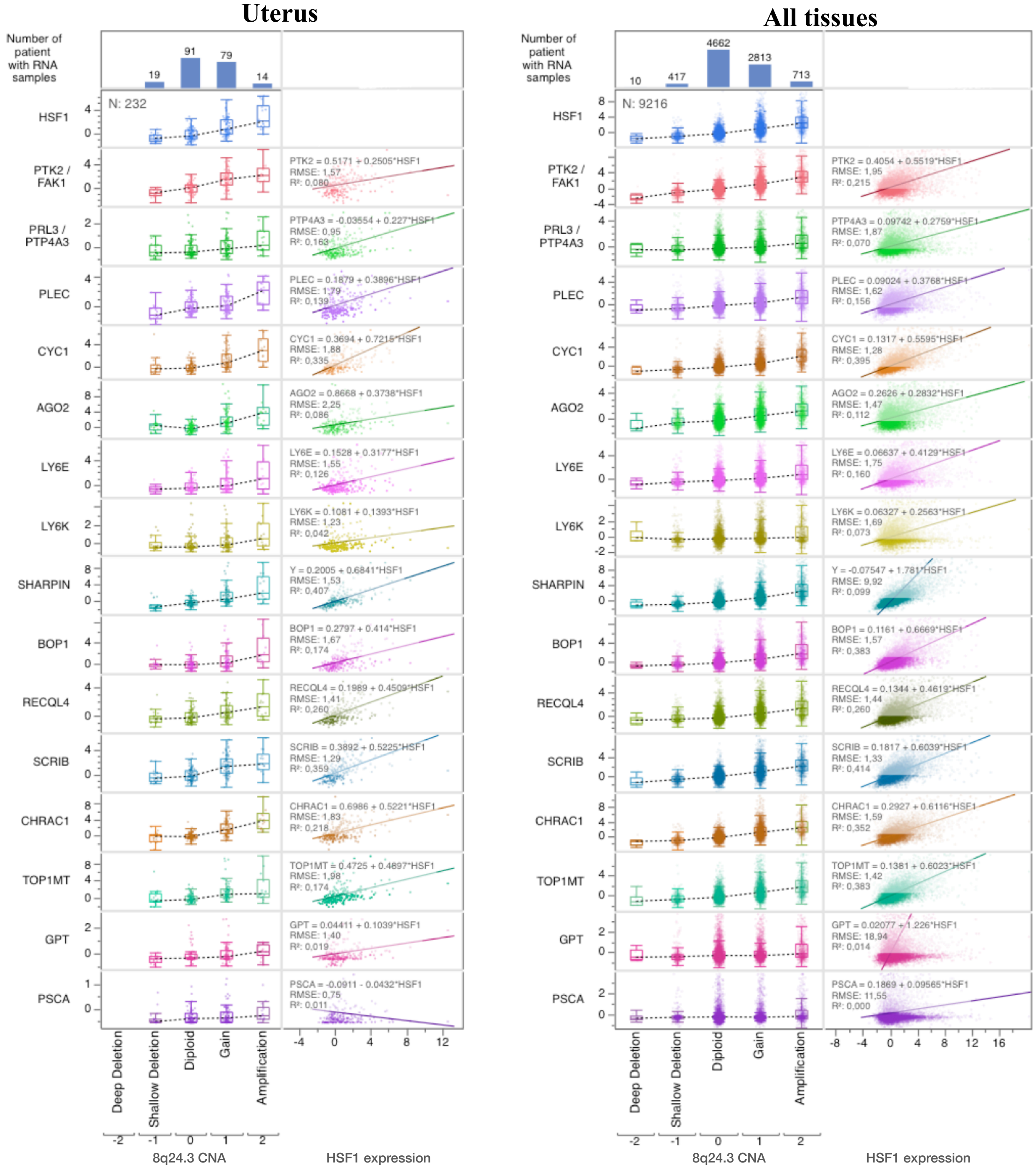

Appendix 5. Influence of 8q24.3 Copy Number on other Cancer-Related Genes Expression Located in 8q24.3 Loci and Comparison with HSF1 Related Expression per Tissue.

Left panel displays the HSF1 RNA expression in function of 8q24.3 copy number. Box plots for each graph of each gene described in **appendix 4** show the median values at the centred lines; box limits indicate the 25th and 75th percentiles; whiskers extend 1.5 times the interquartile range from the 25th and 75th percentiles. Dashed lines rely medians of each copy number category. N = number of patient in each 8q24.3 copy number alteration category are shown in bar graph in top left panel. Right panel displays the RNA expression of the indicated gene in function of HSF1 RNA expression. Each dot represents a patient data. Linear trends are indicated and linear equations are displayed in the caption box for each gene. RMSE = root-mean-square error, R^2 = determination coefficient.

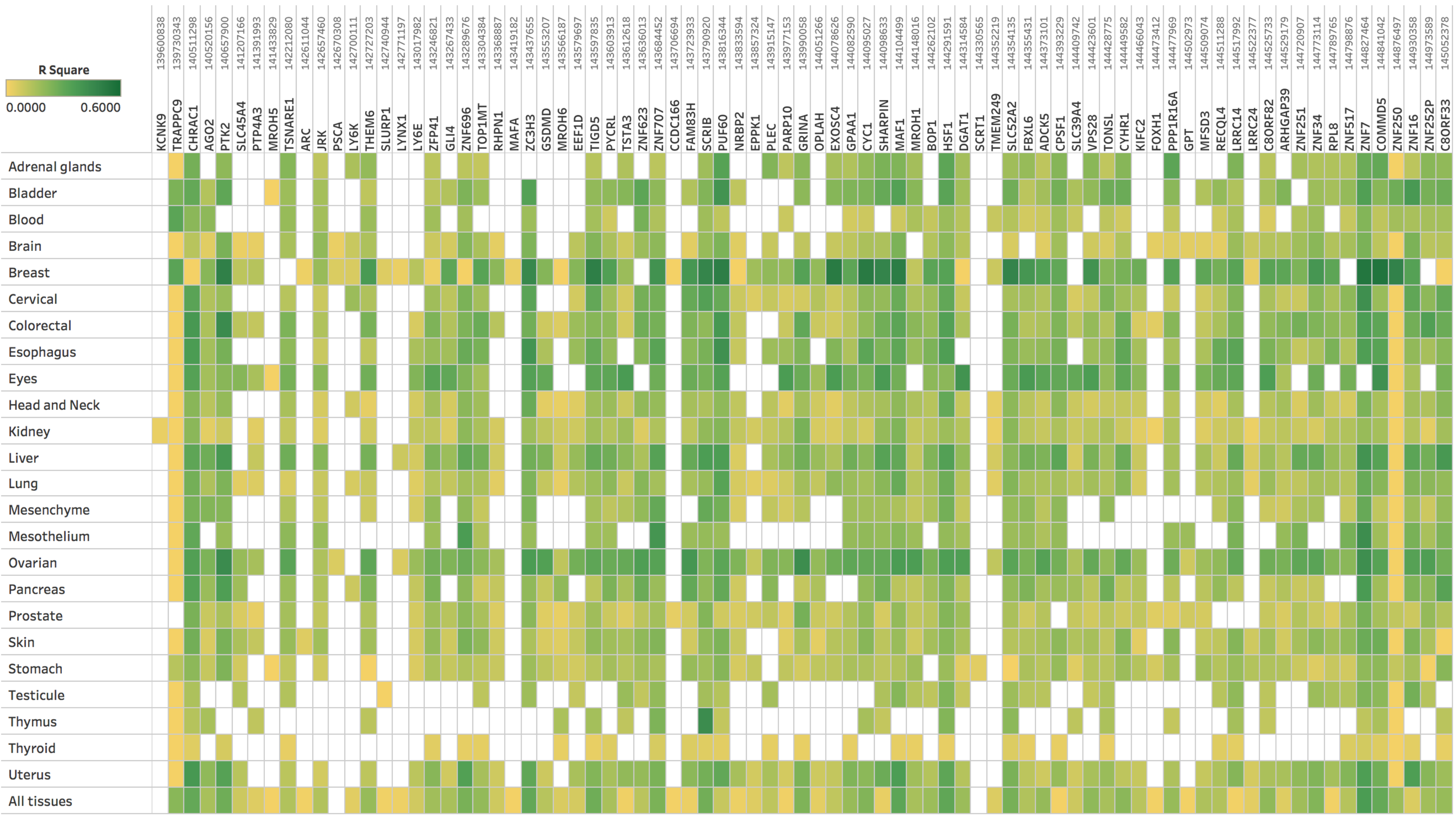

Appendix 6. Simple linear regressions of predicted 8q24.3 CNA compared to the expression of genes located in 8q24.3 per tissue. The heatmap presents simple linear regressions of predicted 8q24.3 CNA (non-heterogenous) compared to the expression of genes located in 8q24.3. The heat map shows genes only located in 8q24.3 with FDR/corrected P-value (Benjamini–Hochberg false discovery rate method) below 0.001. Color legend indicates the calculated R-squared (Explained variation / Total variation). R-square values close to 0 indicates that the gene expression is not correlated with 8q24.3 CNA. R-square values close to 1 indicates that the gene expression is correlated with 8q24.3. Blank squares in the table indicate no available or non-significant data (i.e. FDR p-value >0.001). Values above gene name indicate the start coordinate of the gene in chromosome 8.
